# A New Classification of *Ficus* Subsection *Urostigma* (Moraceae) Based on Four Nuclear DNA Markers (ITS, ETS, G3pdh, and ncpGS), Morphology and Leaf Anatomy

**DOI:** 10.1371/journal.pone.0128289

**Published:** 2015-06-24

**Authors:** Bhanumas Chantarasuwan, Cornelis C. Berg, Finn Kjellberg, Nina Rønsted, Marjorie Garcia, Claudia Baider, Peter C. van Welzen

**Affiliations:** 1 Naturalis Biodiversity Center, Botany, Leiden, The Netherlands; 2 Thailand Natural History Museum, National Science Museum, Pathum Thani, Thailand; 3 Institute Biology Leiden, Leiden University, Leiden, The Netherlands; 4 CEFE UMR 5175, CNRS—Université de Montpellier—Université Paul-Valéry Montpellier–EPHE, Montpellier, France; 5 Natural History Museum of Denmark, University of Copenhagen, Copenhagen, Denmark; 6 Mauritius Herbarium, Reduit, Mauritius; The National Orchid Conservation Center of China; The Orchid Conservation & Research Center of Shenzhen, CHINA

## Abstract

*Ficus* subsection *Urostigma* as currently circumscribed contains 27 species, distributed in Africa, Asia, Australia and the Pacific, and is of key importance to understand the origin and evolution of *Ficus* and the fig-wasp mutualism. The species of subsection *Urostigma* are very variable in morphological characters and exhibit a wide range of often partly overlapping distributions, which makes identification often difficult. The systematic classification within and between this subsection and others is problematic, e.g., it is still unclear where to classify *F*. *amplissima* and *F*. *rumphii*. To clarify the circumscription of subsection *Urostigma*, a phylogenetic reconstruction based on four nuclear DNA markers (ITS, ETS, G3pdh, and ncpGS) combined with morphology and leaf anatomy is conducted. The phylogenetic tree based on the combined datasets shows that *F*. *madagascariensis*, a Madagascan species, is sister to the remainder of subsect. *Urostigma*. *Ficus amplissima* and *F*. *rumphii*, formerly constituting sect. *Leucogyne*, appear to be imbedded in subsect. *Conosycea*. The result of the phylogenetic analysis necessitates nomenclatural adjustments. A new classification of *Ficus* subsection *Urostigma* is presented along with the morphological and leaf anatomical apomorphies typical for the clades. Two new species are described ─ one in subsect. *Urostigma*, the other in *Conosycea*. One variety is raised to species level.

## Introduction

Despite substantial effort, the origin and evolution of *Ficus* L. and the fig-wasp mutualism remain unclear due to lack of resolution of the backbone phylogeny of *Ficus*[1,2,3]. One of the key clades of uncertain placement is *Ficus* subsection *Urostigma*[4,5]. *Ficus* subg. *Urostigma* sect. *Urostigma* subsect. *Urostigma* (Gasp.) C.C. Berg includes 27 species as currently circumscribed with *Ficus religiosa* L. as the type. The distribution of the subsection ranges from West Africa and Madagascar via the Asian mainland to Japan and through (southern) Malesia to Australia and the Pacific. Typical characters of subsect. *Urostigma* are: Trees, many of which are hemi-epiphytic and some terrestrial, aerial roots not abundant, usually intermittent growth, leaves often deciduous, spirally arranged, and articulate or subarticulate (some Asian and African-Madagascan species lack the articulation), inflorescences often borne below the leaves and in some species they are borne on the spurs of the older branches, the colour of the syconium can change from whitish to pinkish, then to purplish, and often finally blackish[6,7]. *Urostigma* was first described in 1844, when Gasparrini[8] broke up the genus *Ficus* into several genera. Later Miquel[9] abandoned this idea and reunited *Ficus*, but divided the genus into six subgenera. Subgen. *Urostigma* was further divided by him into series based on distribution, six series for species in Asia and Australia, three series for African species, and five series for species in America. The species presently included in subsect. *Urostigma* were mainly placed in series *Infectoriae* Miq. and *Religiosae* Miq. of Asia and Australia. The African representatives of the subsection were classified in series *Grandiores* Miq., *Oblongifoliae* Miq., and *Ellipticifoliae* Miq. Later, morphological characters were used to classify the genus, e.g. King [10] divided *Ficus* into seven sections based on leaf morphology. Corner [11] used the colour of the ovary and lithocyst position for his classification, an idea shared by Berg [4]. However, the concept of the sections varied between Corner’s [11] and Berg’s [4] classifications. Berg[4] expanded Corner’s section *Urostigma* by including former sections *Conosycea* and *Leucogyne*[12] and Corner’s concept of sect. *Urostigma* was consequently reduced to the status of subsection. The relationship of the two species(*F*. *amplissima*J.E.Sm.and *F*. *rumphii* Blume) of former sect. *Leucogyne* was questioned when Rønsted et al. [2] published a molecular phylogenetic hypothesis, which showed that *F*. *rumphii* belongs to subsect. *Conosycea*(Miq.) C.C.Berg (*F*. *amplissima* was not included in their study).

At present, molecular phylogenetic analyses have become the major basis for improving classifications. In an early molecular study of *Ficus* by Weiblen[13] using the ITS marker together with morphological data, only three species of *Ficus* subsect. *Urostigma* (*F*. *prasinicarpa* Elmer ex C.C.Berg, *F*. *superba* (Miq.) Miq. and *F*. *virens* Aiton) were included. This study was the first to suggest that the monoecious subgen. *Urostigma* (Gasp.) Miq. was not monophyletic, because sect. *Urostigma* (Gasp.) Endl. appeared to be the sister clade of a functionally dioecious clade, but support for this relationship was weak. Jousselin et al.[14] combined ITS and ETS markers to construct the phylogenetic relationships of 41 species of *Ficus*, including three other species of subsect. *Urostigma* (*F*. *prolixa* G. Forst., *F*. *religiosa* L., and *F*. *salicifolia* Vahl). Their results again suggested that subsect. *Urostigma* forms a separate group from the remainder of subgen. *Urostigma*. Rønsted et al. [2] also combined ITS and ETS in their work, which included nine species of subsect. *Urostigma* and *F*. *rumphii* of sect. *Leucogyne*.Their results indicated that *Ficus* subsect. *Urostigma* is monophyletic when *F*. *rumphii* is excluded (the latter to be transferred to sect. *Conosycea*), and when subsect. *Urostigma* is separated from the rest of subgen.*Urostigma*. Addition of other nuclear markers and more species to the global analysis of *Ficus* have subsequently confirmed a narrow concept of subsect. *Urostigma* excluding *F*. *rumphii*[1,3,15]. However, more than half of the species of subsect. *Urostigma* and *F*. *amplissima* of (former) sect. *Leucogyne* are not included in any phylogenetic analysis yet, thus the monophyly and circumscription of the group is still far from clear.

To solve the problem of the classification of *Ficus* subsect. *Urostigma* and closely related subsections, we began a revision of *Ficus* subsect. *Urostigma*[5] in its traditional classification, congruent with that of Berg[4]. However, we realised that morphology alone did not provide typical characters or a typical combination of charactersto solve the classification problem. Leaf anatomy [16] appeared to show more consistent characters and less variation within species than the morphological characters previously studied [see identification key in 5] and, especially when combined with morphology, leaf anatomical characters provided a highly accurate tool for species recognition, enabling recognition of some of the morphologically highly variable species (e.g., *F*. *virens*). Leaf anatomical evidence also suggested that *F*. *amplissima* more closely resembles *F*. *arnottiana* (Miq.) (subsection *Urostigma*) Miq. than *F*. *rumphii*(former sect. *Leucogyne*). A result that contradicted the classification presented in[4,5].

Therefore, the main aims of this study are (1) to create a comprehensive phylogenetic hypothesis of subsect.*Urostigma* by analysing several molecular markers (ITS, ETS, G3pdh, and ncpGS) for almost all known species of subsect. *Urostigma* and related groups, and (2) to propose a new classification of subsect. *Urostigma* based on the resulting phylogenetic hypothesis.

## Materials and Methods

### Taxon sampling

In total, 76 taxa were represented corresponding to 36 species out of c. 280 spp. of *Ficus* subgen. *Urostigma*, including 24 out of 27 species of subsect. *Urostigma*, and five (out of 60) species representing *Urostigma* subsect. *Conosycea* (*F*. cf. *rumphii*, *F*. *altissima* Blume, *F*. *benjamina* L., *F*. *glaberrima* Blume subsp. *siamensis* (Corner) C.C. Bergand *F*. *menabeensis* H. Perrier), as well as two species from each of sect. *Americana*(*F*. *americana* Aubl., *F*. *aurea* Nutt.; c. 100 species), sect. *Stilpnophyllum* subsect. *Malvanthera*(*F*. *pleurocarpa* F.Muell., *F*. *brachypoda* (Miq.) Miq.; c. 20 species), one species of sect. *Leucogyne* (*F*. *rumphii*), and one species of sect. *Galoghycia*(*F*. *bubu*Warb.; c. 72 species). Two species of subgen. *Pharmacosycea*(*F*. *maxima* Mill. and *F*. *tonduzii* Standl.) were included as outgroup representing the first diverging lineage of *Ficus* as currently understood [1].

Dried leaf samples from 37 herbarium collections and 26 leaf samples dried on silica gel were used for DNA extraction(for voucher information see S1 Appendix). The silica gel samples together with vouchers were collected in non-protected areas for the access of which no permits were needed (no specific permissions were required for these locations/activities and the field studies did not involve endangered or protected species); see Table 1 for localities. The species involved are non-CITES protected. DNA sequence data were sampled for four nuclear DNA markers (ITS, ETS, G3pdh, ncpGS). In total, 234 sequences were used in the analysis, including 199 new sequences and 35 sequences downloaded from GenBank. All new sequences are available from GenBank (S1 Appendix).

**Table 1:** Locations where silica dried samples were taken.

Subsection	Species	Vouchers	Location
*Urostigma*	*Ficus caulocarpa* (Miq.) Miq.	Chantarasuwan 261111-1(L)	Thailand, Trang, Nayong, 7°32'47.3"N 99°45'41.9"E
	*Ficus caulocarpa* (Miq.) Miq.	Chantarasuwan 071010–2 (L)	Thailand, Nakhon Si Thammarat, Noppitam, 8°44'32.5"N 99°44'18.8"E
	*Ficus concinna* (Miq.) Miq.	Chantarasuwan 071010–1 (L)	Thailand, Nakhon Si Thammarat, Thasala, 8°32'54.0"N 99°56'27.3"E
	*Ficus concinna* (Miq.) Miq.	Chantarasuwan 140910–3 (L)	Thailand, Ratchaburi, Chombung, 13°35'29.6"N 99°40'15.3"E
	*Ficus concinna* (Miq.) Miq.	Chantarasuwan 120910-5(L)	Thailand, Rayong, Pe, 12°36'57.0"N 101°24'52.9"E
	*Ficus concinna* (Miq.) Miq.	Chantarasuwan 051010-4(L)	Thailand, PrachuapKhiri Khan, Kuiburi,12°12'23.3"N 100°00'32.2"E
	*Ficus geniculata* Kurz var. *geniculata*	Chantarasuwan 150910–1 (L)	Thailand, Kanchanaburi, Thong PhaPhum, Lintin, 14°31'55.4"N 98°48'43.2"E
	*Ficus geniculata* Kurz var. *geniculata*	Chantarasuwan 210910–1 (L)	Thailand, Lamphun, Muang, 18**°**40'11.7"N 99**°**03'20"E
	*Ficus geniculata* Kurz var. *geniculata*	Chantarasuwan 301111–1 (L)	Thailand, Chiang Rai, Muang, Pongsali, 19°50'25.3"N 99°47'33.7"E
	*Ficus middletonii* Chantaras.	Chantarasuwan 051010–2 (L)	Thailand, Prachuap Khiri Khan, Kuiburi, 12°12'24.5"N 100°00'31.1"E
	*Ficus orthoneura* H.Lév. &Vaniot	Chantarasuwan 231111–1 (L)	Thailand, Tak, Phobpra, 16°34'39.4"N 98°38'57.6"E
	*Ficus religiosa* L.	Chantarasuwan 110910–4 (L)	Thailand, Sa Kaeo, Khao Chakan, 13°39'29.3"N 102°04'58.9"E
	*Ficus religiosa* L.	Chantarasuwan 150910–2 (L)	Thailand, Kanchanaburi, Thong PhaPhum, Lintin, 14**°**29'44.6"N 98**°**50'20.2"E
	*Ficus subpisocarpa* Gagnep. subsp. *pubipoda* C.C. Berg	Chantarasuwan 110910–1 (L)	Thailand, Chachoengsao, Panom Sarakham, Khao Hin Son, 13°45'48.5"N 101°30'51.8"E
	*Ficus subpisocarpa* Gagnep. subsp. *pubipoda* C.C. Berg	Chantarasuwan 011211–1 (L)	Chachoengsao, Panom Sarakham, Khao Hin Son, 13**°**45'42.5"N 101**°**30'51.8"E
	*Ficus superba* (Miq.) Miq.	Chantarasuwan 120910–2 (L)	Thailand, Rayong, Kleang, 12°41'15.0"N 101°37'57.8"E
	*Ficus virens* Aiton var. *glabella* (Blume) Corner	Chantarasuwan 071010–3 (L)	Thailand, Nakhon Si Thammarat, Noppitam, Krung Ching, 8°47'47.2"N 99°38'00.7"E
	*Ficus virens* Aiton var. *glabella* (Blume) Corner	Chantarasuwan 071010–4 (L)	Thailand, Nakhon Si Thammarat, Noppitam, Krung Ching, 8°48'18.4"N 99°36'39.5"E
*Conosycea*	*Ficus glaberrima* Blume subsp. *siamensis* (Corner) C.C.Berg	Chantarasuwan 110910–2 (L)	Thailand, Sa Kaeo, Khao Chakan, Wat Khao Chakan, 13°39'37."N 102°05'06.4"E
	*Ficus glaberrima* Blume subsp. *siamensis* (Corner) C.C.Berg	Chantarasuwan 110910–3 (L)	Thailand, Sa Kaeo, Khao Chakan, Wat Khao Chakan,13°39'32.9"N 102°04'54.5"E
	*Ficus glaberrima* Blume subsp. *siamensis* (Corner) C.C.Berg	Chantarasuwan 180910–3 (L)	Thailand, Lop Buri, Thawung, Wat Khao Samorkorn, 14**°**53'59.9"N 100**°**30'42.9"E
	*Ficus* cf. *rumphii* Blume	Chantarasuwan 180910–2 (L)	Thailand, Lop Buri, Thawung, Wat Khao Samorkorn, 14°54'07.9"N 100°30'32.5"E
	*Ficus rumphii* Blume	Chantarasuwan 120910–4 (L)	Thailand, Rayong, Pe, 12°37'01.9"N 101°24'48.5"E
	*Ficus rumphii* Blume	Chantarasuwan 140910–1 (L)	Thailand, Ratchaburi, Chombung, 13°35'27.0"N 99°40'14.5"E

### DNA extraction, amplification, and sequencing

About 20–50 mg of dried leaf tissue from each sample was used for extraction using the Qiagen DNeasy Plant Kit and following the manufacturer’sprotocol. We sequenced the nuclear encoded ITS, ETS, G3pdh and ncpGS regions following protocols in previous studies [1, 2, 3, 17, 18]. The primer sequences for all markers are shown in Table 2. The Polymerase chain reactions (PCR) were performed with 1μL of DNA product, 10 μL of Red-Sigma buffer (Qiagen Inc.), 2μL of each 10 μM primers(forwardand reverse), 0.4 μL of BSA (Promega, Madison, Wisconsin, USA) and 6.6 μL of H_2_O, in a total volume of 20 μL. The PCR programmes followed are summarised in Table 3.PCR fragments were checked for length and yield by gel electrophoresis on 2% agarose gels and cleaned using the Qiagen PCR clean-up kit before sequencing on an ABI 377 Genetic Analyzer according to the manufacturer's protocols (Applied Biosystems). Both strands were sequenced for each region for the majority of taxa.

**Table 2:** Sequences of primers used in this study.

Region	Primer sequence	Reference
ITS	ITS_5F: 5´-GGA AGT AAA AGT CGT AAC AAG G-3´, ITS_4R: 5´-TCC TCC GCT TAT TGA TAT GC-3´, ITS_17SE: 5´-ACG AAT TCA TGG TCC GGT GAA GTG TTC G-3´, ITS_26SE: 5´-TAG AAT TCC CCG GTT CGC TCG CCG TTA C-3´	[38], [38], [17], [17]
ETS	ETS_Hel1: 5´-GCT CTT TGC TTG CGC AAC AAC T-3´, 18S_ETS: 5´-GCA GGA TCA ACC AGG TAG CA- 3´, ETS_Fig1_F: 5´-GACCCTTGGTTCCTGTGTTGC-3´	[39], [39], [Bruun-Lund & Rønsted, unpublished]
G3pdh	GPDX7F: 5´-GAT AGA TTT GGA ATT GTT GAG G-3´, GPDX9R: 5´-AAG CAA TTC CAG CCT TGG-3´	[18], [18]
ncpGS	GS_3F: 5´-GTT GTG ATT WAC CAT GCT-3´, GS_4R: 5´-AGA TTC AAA ATC GCC TTC-3´	[1], [1]

Notes: The primer combinations ITS_5F plus ITS 4R and ITS17SE plus ITS26SE were used interchangingly with about equal success corresponding to standard protocols at C and CNRS. The combination of the *Ficus* specific internal primer ETS_Fig1_F plus 18S_ETS was only used for amplification of 13 accessions across the subsection, which could not be amplified with the standard primers.

**Table 3:** PCR programsadjusted from [1,2,3] as used for each molecular marker.

Regions	PCR program
ITS	2 min. at 94°C followed by 35 cycles of 30 sec. denaturation (94°C), 1 min. annealing (63°C), and 1 min. extension (72°C) and 10 cycles of 30 sec. denaturation (94°C), 1 min. annealing (60°C), and 1 min. extension (72°C). After the last cycle, the temperature was kept at 72°C for a final 5 min. extension and then lowered to 16°C.
ETS	2 min. at 94°C followed by 45 cycles of 30 sec. denaturation (94°C), 1 min. annealing (60°C), and 1 min. extension (72°C). After the last cycle, the temperature was kept at 72°C for a final 5 min. extension and then lowered to 16°C.
G3pdh	2 min. at 94°C followed by 40 cycles of 30 sec. denaturation (94°C), 1 min. annealing (62°C), and 1 min. extension (72°C) and 10 cycles of 30 sec. denaturation (94°C), 1 min. annealing (56°C), and 1 min. extension (72°C). After the last cycle, the temperature was kept at 72°C for a final 5 min. extension and then lowered to 16°C.
ncpGS	2 min. at 94°C followed by 45 cycles of 30 sec. denaturation (94°C), 1 min. annealing (57°C), and 1 min. extension (72°C) After the last cycle, the temperature was kept at 72°C for a final 5 min. extension and then lowered to 16°C.

### DNA sequence alignments

Sequences were initially edited and improved by eye using CodonCode Aligner (CodonCode Corporation, Dedhem, USA) and MacClade 4.08 OSX[19], and both forward and reverse sequences were assembled. All assembled sequences were blasted via GenBank database to check for possible contamination with non-*Ficus* DNA. The alignment of whole sequences was done online with Phylogeny.fr, option MUSCLE[20], and SeaView 3.2[21]. Gaps were treated as missing data and indels were excluded from the alignments, because they were not informative or only supported clades that already received high support. Missing markers were also coded as missing data.

### Morphological and leaf anatomical data

The morphological data matrix was constructed using the most recent taxonomic revision of *Ficus* subsection *Urostigma*[5]. The specimens used in the revision were also the primary source for compiling the data matrix. In addition, specimens, stored in L, representing the species from other infrageneric taxa were also used to score data. In total, 43 qualitative morphological characters were coded for analysis (see S2 Appendix for characters, and S3 Appendix for the data matrix). The leaf anatomical data are based on recent work by Chantarasuwan et al.[16], to which the character states of non-subsect. *Urostigma* species were added, either studied (*F*. cf.*rumphii*) or extracted from Berg and Corner [7]. In total 23 qualitative characters were coded for analysis (see S2 Appendix for characters, and S3 Appendix for the data matrix). All characters were treated as unordered and of equal weight, missing data were coded as unknown. Characters 8, 9, 11, 12, 13, 18, 21, 33, 34, 37, 45, and 63 are in fact continuously distributed. However, these characters are coded as having discrete states, because several characters show a gap (9, 18, 21, 45) or a soft gap (all others), whereby the few taxa with overlap are coded as polymorphic (both states present).

### Phylogenetic analysis

In total five analyses were made. The analyses of the four combined molecular DNA markers were performed with Maximum Parsimony (MP) and Bayesian Inference (BI) methods. The morphology and leaf anatomy dataset was analysed under Maximum Parsimony (MP). Both datasets, molecular and morphology/leaf anatomy, were subsequently combined (total evidence approach) and analysed under MP and BI.

The MP analyses were run using PAUP* v4.0b10 [22] and heuristic searches with 3000 replicates,ten random taxon additions, tree-bisection-reconnection branch swapping (TBR), MulTrees option active, and no more than 10 trees saved per replicate. Branch support was performed in PAUP with bootstrap analyses [23] with 1000 replicates and all other settings similar to the phylogeny analysis. Bootstrap percentages(BS) are defined as high (85–100%), moderate (75–84%), low (50–74%) or no support (<50%).

Model selection for the Bayesian analysis was conducted using the model selection tool available through the online HIV sequence database site (http://www.hiv.lanl.gov/content/sequence/findmodel/findmodel.html) checking all 28 models and constructing the initial tree with Weighbor (default)[22]. The chosen models were HKY+G for ITS, GTR+G for ETS, HrN+G for G3pdh, and HKY+G for ncpGS (JC for the morphological and anatomical data after the manual of MrBayes [24]). The datasets were analysed online using MrBayes v.3.1.2[24] with 100,000,000 generations via the Cipres science gateway(http://www.phylo.org). The default values of 4 chains (3 heated, 1 cold, temperatures default) and two parallel runs were used, whereby every 1,000^th^ cladogram was sampled. A 10% burn-in was executed after Tracer 1.6 [25] was used for each tree file to check whether or not the effective sampling sizes (ESS) of all parameters exceeded 200, indicating that they are a good representation of the posterior distributions. The Potential Scale Reduction Factors (PSRF) in the MrBayes SUMP output were 1 or close to 1, which also indicates correct convergence. Bayesian inference produces posterior probabilities that are relatively higher than the corresponding bootstrap frequencies [26], thus we only used posterior probabilities (PP) above 0.9 as (high) support. TreeAnnotator v.1.8.0 (part of BEAST v.1.8.0 package [27,28]) was used to create a Maximum Clade Credibility (MCC) tree from every run. These did not differ in topology, only somewhat in support. The MCC tree of the first run was selected.

Mesquite v.2.7.5 [29] was used to show the changes in morphological and anatomical characters on the MCC tree from the Bayesian analysis of the combined datasets (see discussion for the preferred MCC tree, the molecular one or the one based on combined data one).

### Nomenclature

The electronic version of this article in Portable Document Format (PDF) in a work with an ISSN or ISBN will represent a published work according to the International Code of Nomenclature for algae, fungi, and plants, and hence the new names contained in the electronic publication of a PLOS ONE article are effectively published under that Code from the electronic edition alone, so there is no longer any need to provide printed copies.

In addition, new names contained in this work have been submitted to IPNI, from where they will be made available to the Global Names Index. The IPNI LSIDs can be resolved and the associated information viewed through any standard web browser by appending the LSID contained in this publication to the prefix http://ipni.org/. The online version of this work is archived and available from the following digital repositories: PubMed Central, LOCKSS.

## Results

### Analysis of DNA datasets

Seventy six taxa were included in the combined dataset with varying amplification success for the four DNA regions targeted as also found previously [1]: 74 taxa provided ITS data, 68 taxa ETS sequences, 53 taxa G3pdh sequences, and only38 taxa provided ncpGS sequences. MP analyses of the separate markers did not show major incongruences in topology, therefore all were united. The combined aligned data matrix was 2674 bp long with 472 potentially informative characters. The MP analysis resulted in 1300 most parsimonious trees (MPTs) with a length = 1636, consistency index (CI) = 0.68, and retention index (RI) = 0.78 (all characters included, informative and uninformative). The strict consensus tree (not shown) of 1300 most parsimonious trees (MPTs) contains two major clades.

**Fig 1:**
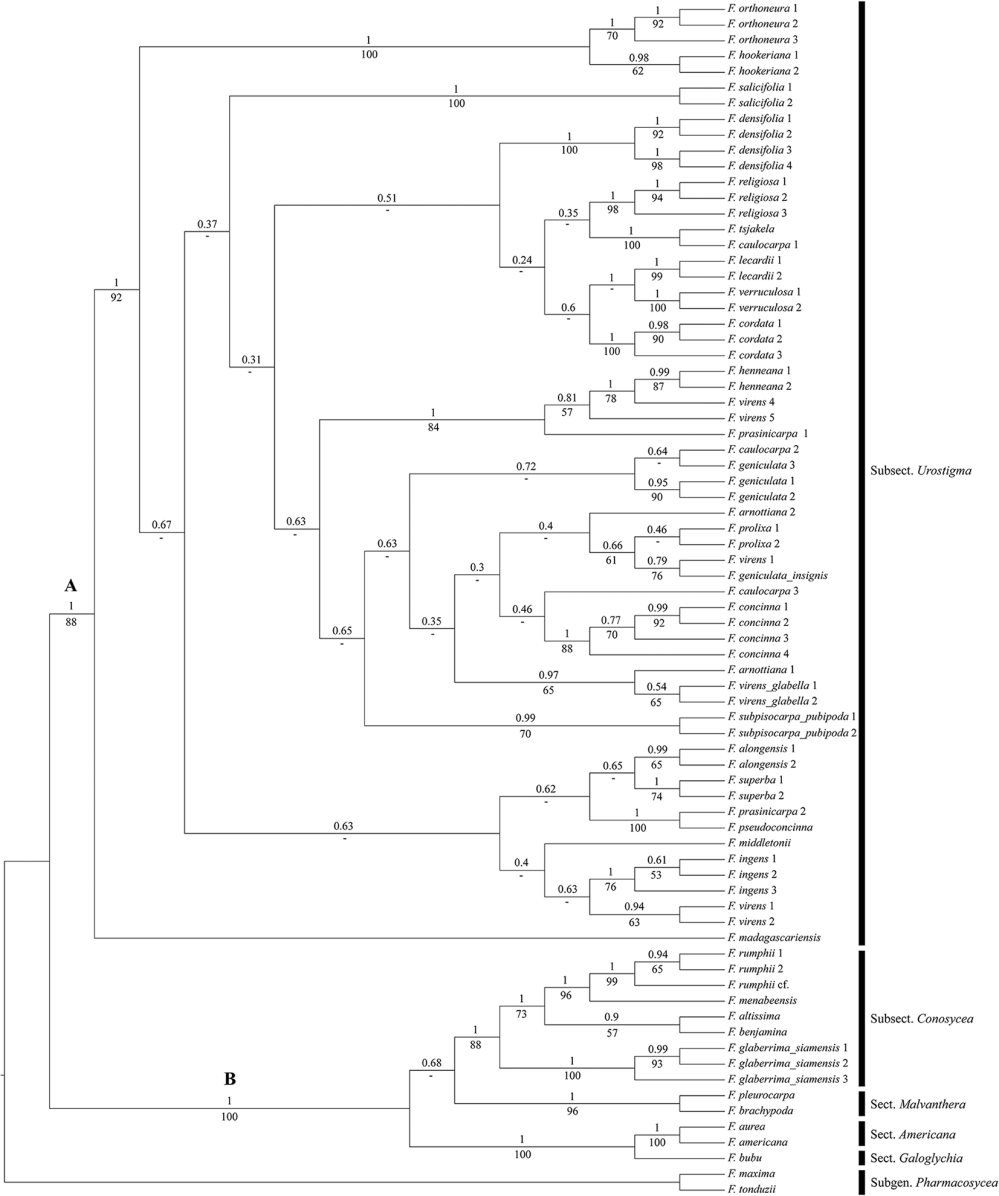
Maximum Clade Credibility (MCC) tree from Bayesian analysis of four combined DNA markers (ITS, ETS, G3pdh, and ncpGS) with posterior probabilities (PP) above and bootstrap supports (BS) below the branches.

The same two clades are present in the MCC tree (Fig 1) of the Bayesian analysis. Clade A comprises all members of subsect. *Urostigma* with a support of BS = 88 and PP = 1. *Ficus madagascariensis* is sister to the rest of this clade (high support, BS = 92 and PP = 1). Within clade A most internal nodes show low support, except for the higher support for most nodes that unite the various specimens of a species. Several of these species are not monophyletic, *F*. *arnottiana*, *F*. *caulocarpa*, *F*. *prasinicarpa*, *F*. *virens* are polyphyletic (different ancestral nodes included), and *F*. *geniculata* is paraphyletic (shared ancestral node, not all descending lineages included). Clade B (BS = 100, PP = 1) contains the members of sect. *Americana*, sect. *Galoghycia*, sect. *Malvanthera*, subsect. *Conosycea*, and *F*. *rumphii* of sect. *Leucogyne*.

### Analysis of morphological and leaf anatomical data

A total of 43 morphological and 23 leaf anatomical characters were used. The MP analysis resulted in 1368 most parsimonious trees with a length = 280, CI = 0.25, and RI = 0.77 (including uninformative characters). The resulting strict consensus tree is a single extended polytomy(not shown).

### Analysis of DNA markers combined with morphology and leaf anatomy

A total of 2740 characters, 2674 molecular (ITS, ETS, G3pdh, and ncpGS) and 66 morphological and leaf anatomical characters were used; of these 538 characters were parsimony informative. The MP analysis resulted in81 most parsimonious trees with a tree length = 1964 (including uninformative characters), CI = 0.5988, and RI = 0.7560 (strict consensus not shown).

**Fig 2:**
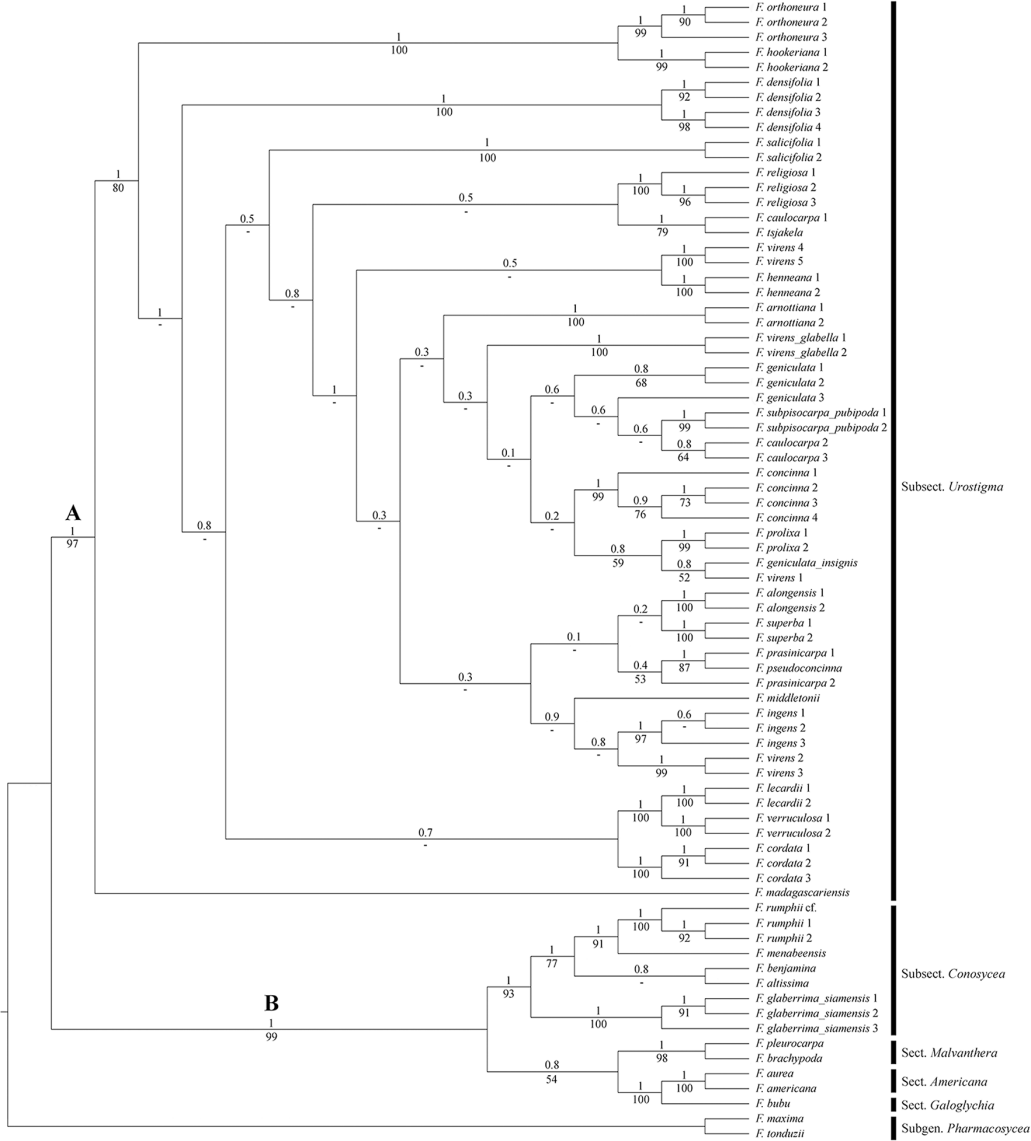
Total evidence MCC tree fromBayesian analysis of four DNA markers, morphology and leaf anatomy. Posterior probabilities (PP) above and bootstrap supports (BS) below the branches.

Tracer [26] showed that all variables in the results of the BI analysis had an effective sampling size far above 200 (326–1851). The MCC tree is shown in Fig 2.

The cladogram (Fig 2) shows the same two distinctive subclades as found in the analysis of the four combined DNA markers (Fig 1). Clade A (BS = 97, PP = 1) is composed of all species of subsect. *Urostigma* with *F*. *madagascariensis* as the first divergent lineage. Similar as with the molecular data analysis (Fig 1), relationships within the remainder of clade A are not well supported in the combined analysis. The species that are represented by several samples usually form monophyletic groups (with high support) except for *F*. *caulocarpa*, *F*. *geniculata*, *F*. *prasinicarpa* and *F*. *virens*. *Ficus prasinicarpa* is paraphyletic because of the inclusion of *F*. *pseudoconcinna*; the clade itself has low support (BS = 53, PP = 0.4), but *F*. *prasinicarpa 2* and *F*. *pseudoconcinna* have high support (BS high = 87, PP high = 1). *Ficus geniculata 3* groups with *F*. *caulocarpa 2* and *3* and *F*. *subpisocarpa* Gagnep. subsp. *pubipoda*, but with very low support (BS<50, PP = 0.6).

Two species, represented by several samples, appear to bepolyphyletic, *F*. *caulocarpa* (Miq.) Miq. and *F*. *virens*. Of the three samples of *F*. *caulocarpa*, *F*. *caulocarpa 1* forms a clade with *F*. *tsjakela* Burm.f. (BS moderate = 79, PP high = 1), while *F*. *caulocarpa2* and *3* form a clade together as described above. Accessions of *F*. *virens* appears in four places; variety *virens* appears in three clades, *F*. *virens 1* groups with *F*. *geniculata* var. *insignis* (low support, BS = 52, PP = 0.8), *F*. *virens 2* and *3* group together (strong support,BS = 99 and PP = 1) and are further linked to the three specimens of *F*. *ingens* (Miq.) Miq., and *F*. *virens4* and *5* group together(strong support, BS = 100 and PP = 1) and further group with two specimens of *F*. *henneana* Miq. The two specimens of *F*. *virens* var. *glabella* (*F*. *virens* var. *glabella* 1 and *F*. *virens* var. *glabella 2*) also form aseparate clade with high support (BS = 100 and PP = 1).

Clade B is composed of members of sect. *Americana*, sect. *Galoghycia*, subsect. *Malvanthera*, subsect. *Conosycea*, and *F*. *rumphii* of sect. *Leucogyne*. Particularly subsect. *Conosycea* is well supported (BS = 93 and PP = 1) and includes the three accessions of *F*. *rumphii*(BS = 100 and PP = 1).

### Character mapping

**Fig 3:**
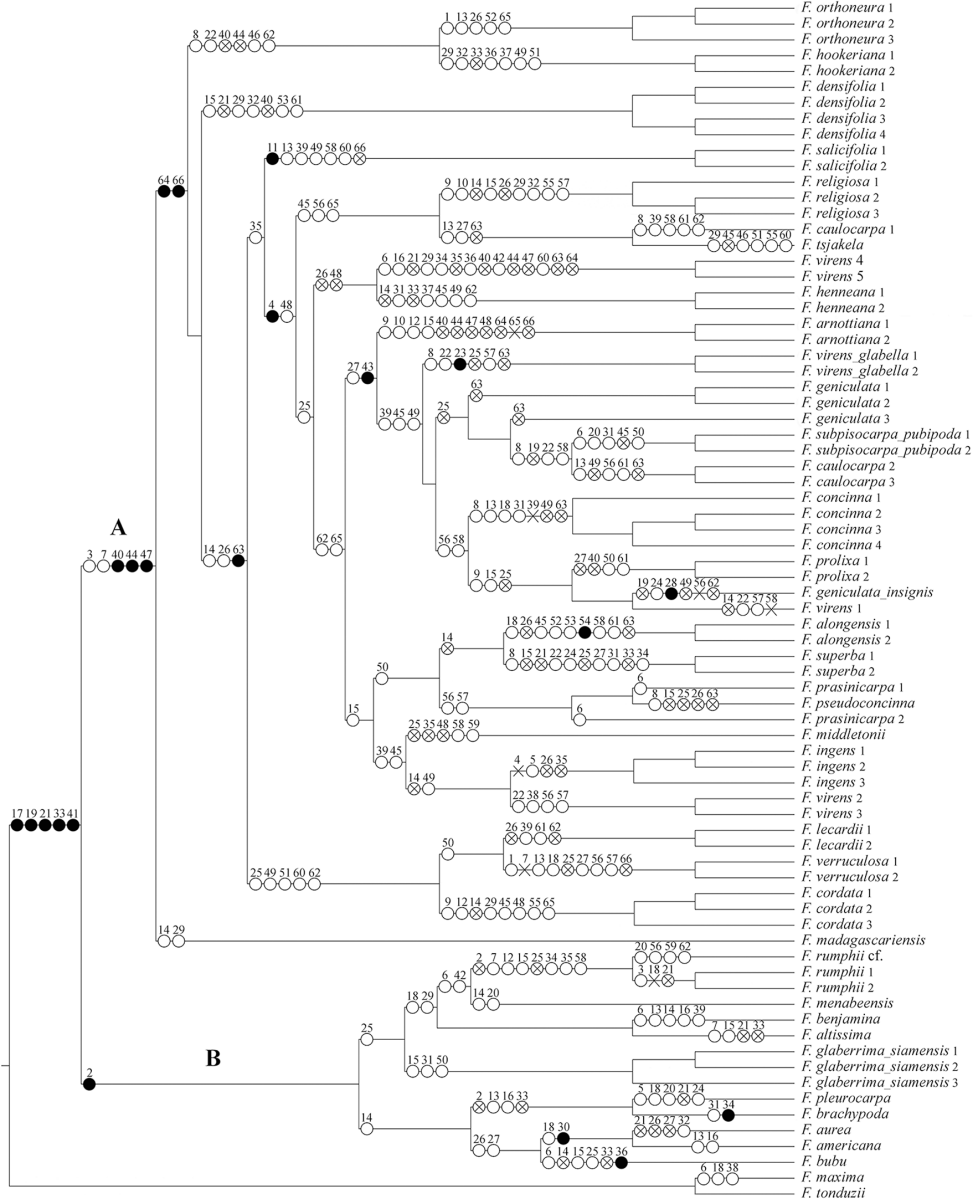
Parsimony distribution of the morphological characters and their states on the total evidence MCC Bayesian tree (Fig 2). ● = unique apomorphy; ○ = parallelism; ✕ = reversal; ⨂ = parallel reversal.

The morphological and leaf anatomical character state changesare summarised in Fig 3. Subsect. *Urostigma*(clade A in Fig 3) is supported by the following apomorphies: intermittent growth(character 3, state 2; shared in parallel with *F*. *rumphii* of subsect. *Conosycea*, clade B), deciduous leaves (char. 7, state 1; reversal in *F*. *verruculosa*, parallel with some species of subsect. *Conosycea*: *F*. *altissima*, *F*. *rumphii* and *F*. cf.*rumphii*), staminate flowers near ostiole (char. 40, state 1; parallel reversals in *F*. *arnottiana*, *F*. *densifolia*, *F*. *hookeriana*, *F*. *orthoneura*, *F*. *prolixa*, and *F*. *virens4* and *5*), single-layered epidermis (char. 44, state 1; parallel reversals in *F*. *arnottiana*, *F*. *virens4* and *5*, *F*. *orthoneura*, and *F*. *hookeriana*), abaxial enlarged lithocysts(char. 47, state 1; parallel reversals in *F*. *arnottiana* and *F*. *virens4* and *5*).

## Discussion

### Phylogenetic circumscription of *Ficus* subsect. *Urostigma*

Our results based on comprehensive sampling of subsection *Urostigma* are consistent with recent previous studies at the genus level supporting a narrow concept of subsect. *Urostigma* s.s. excluding former sect. *Leucogyne*[1,2,3,15]. Unfortunately the extraction of DNA from *F*. *amplissima*, the other species of sect. *Leucogyne*, was unsuccessful in our study, but a partial ITS sequence of *F*. *amplissima*(Rønsted, unpublished; specimen Matthew 20582 (K)) forms a clade together with *F*. *rumphii* embedded in the *Conosycea* clade. This is supported by evidence from the pollinators, because *F*. *amplissima* and *F*. *rumphii* are pollinated by the same wasp genus (*Eupristina*), a genus only known to be associated with species of subsect. *Conosycea*[7,30], which is indicative of co-evolution [1]. Based on these two independent pieces of evidence were classify *F*. *amplissima* in subsection *Conosycea*, which means that the complete sect. *Leucosyce* should now be synonymised with subsect. *Conosycea*. Corner[31] originally considered *F*. *prolixa*, a Polynesian species, to be related to the American hemi-epiphytic figs of sect. *Americana*, because of the scattered position of the staminate flowers in the fig. However, *F*. *prolixa* has three basal bracts and not two as in sect. *Americana*. Our phylogenetic results clearly show that there is no close relation between *F*. *prolixa*(clade A) and sect. *Americana* (clade B).

Relationships within subsection *Urostigma* s.s. are still not well supported based on four nuclear genes, morphology and leaf anatomy, and further work (e.g., with massive parallel sequencing) is needed before subdivision of the subsection.

### Molecular versus Total Evidence

Fig 1 (molecular data only) and Fig 2 (molecular and morphological/leaf anatomical data) show both two major clades, A (subsect.*Urostigma*) and B (other (sub)sections), with in clade A *F*. *madagascariensis* as basal lineage, followed by the lineage *F*. *orthoneura*-*F*. *hookeriana*. All other clades are often the same in Figs 1 and 2, but they differ in sister group relations. The clade *F*. *lecardii-F*. *verruculosa-F*. *cordata* is similar in both phylogenies, but is more basal in Fig 2 than in Fig 1. The clade *F*. *alongensis-F*. *virens3* is again in a different position, but *F*. *prasinocarpa 1* is not part of the clade in Fig 1. In clade 1 *F*. *prasinocarpa 1* is sister to a clade with a paraphyletic *F*. *virens* (*4*&*5*) and a monophyletic *F*. *henneana*; in Fig 2 *F*. *virens4 & 5* are monophyletic and sister to the *F*. *henneana* specimens. The differences were to be expected considering the low support for the internal branches of clade A in Figs 1 and 2. The differences between and low support in both analyses precludes an infrageneric classification.

In the cladogram from the combined, total evidence approach (Fig 2), the species with multiple samples are more often grouped together than in the molecular phylogeny (Fig 1), (specimens *F*. *arnottiana*, *F*. *prasinicarpa*(paraphyletic) grouped together in Fig 2, both polyphyletic in Fig 1). Moreover, Fig 2 provides a much better historical biogeographic scenario than Fig 1 (not elaborated here); for instance the Madagascan and African taxa group are more grouped together and basal in Fig 2 than in Fig 1 (*F*. *madagascariensis*, *F*. *cordata*, *F*. *densifolia*, *F*. *lecardii*, *F*. *salicifolia*, *F*. *verruculosa*). In general, the support, especially in the terminal branches, is much higher in the total evidence approach (Fig 2) than in the molecular analysis (Fig 1). Based on these three reasons we prefer the results of the total evidence approach (Fig 2) above the results of the molecular data only (Fig 1). This conclusion supports the idea of Wiens [32] that morphology and leaf anatomy add valuable data to the phylogeny reconstruction when combined with molecular data.

### Comparing the phylogeny with traditional classifications

To some degree, our phylogenetic results support the geographical implications of the classification made by Miquel[9], with the taxa arranged per continent (e.g., a group of African species separate from Asian species). However, there are a few exceptions. In our results(Fig 1) one African species, *F*. *ingens*, is placed among Asian species, and Sino-Himalayan *F*. *hookeriana* and *F*. *orthoneura* are among African species. Thus, a purely continental classification is not attainable. Corner[12,33] divided sect. *Urostigma* (similar to subsect. *Urostigma* here) of Asia and Australia into four series, *Religiosae* Miq., *Superbae* Corner, *Caulobotryae* (Miq.) Corner, and *Orthoneurae* Corner. However, species in the various series of Corner do not form monophyletic groups, but are mixed in our phylogenetic tree and the relationships among clades are not well supported. Moreover, Corner never included the African species, precluding direct comparison with his subdivision. Berg[4] re-classified sect. *Urostigma* and included African species, only recognising two subsections, *Urostigma* and *Conosycea*, and no series. Berg’s classification compares well with ours and previous work [1,2,3,15] results of two clades, which cannot easily be subdivided into recognisable subgroups (low support for most branches and no distinct character combinations in Fig 3). Berg included *F*.*amplissima* and *F*. *rumphii* (formerly in *Leucosyce*) in subsect. *Urostigma*, which is not consistent with our results, which point at inclusion in subsect. *Conosycea* (see below).

### Homoplasy in characters used or suitable for recognising subsect. *Urostigma*

The character mapping showed three unique apomorphies for the subsect. *Urostigma* clade (Fig 3), one morphological character (40.1: staminate flowers near ostiole), and two leaf anatomical characters (44.1: epidermis simple; 47.1: enlarged lithocysts only abaxially). Two morphological characters (3.1: intermittent growth present; 7.1: leaves deciduous) show parallel apomorphies in *Conosycea*, though the combination is unique. All characters were previously used for the recognition of subsect. *Urostigma*by [4,5,7]. These resultsimply that the morphological data used here are not sufficient to separate both subsections, whereas the combination with leaf anatomy allows a distinct subsectional recognition.

**Fig 4:**
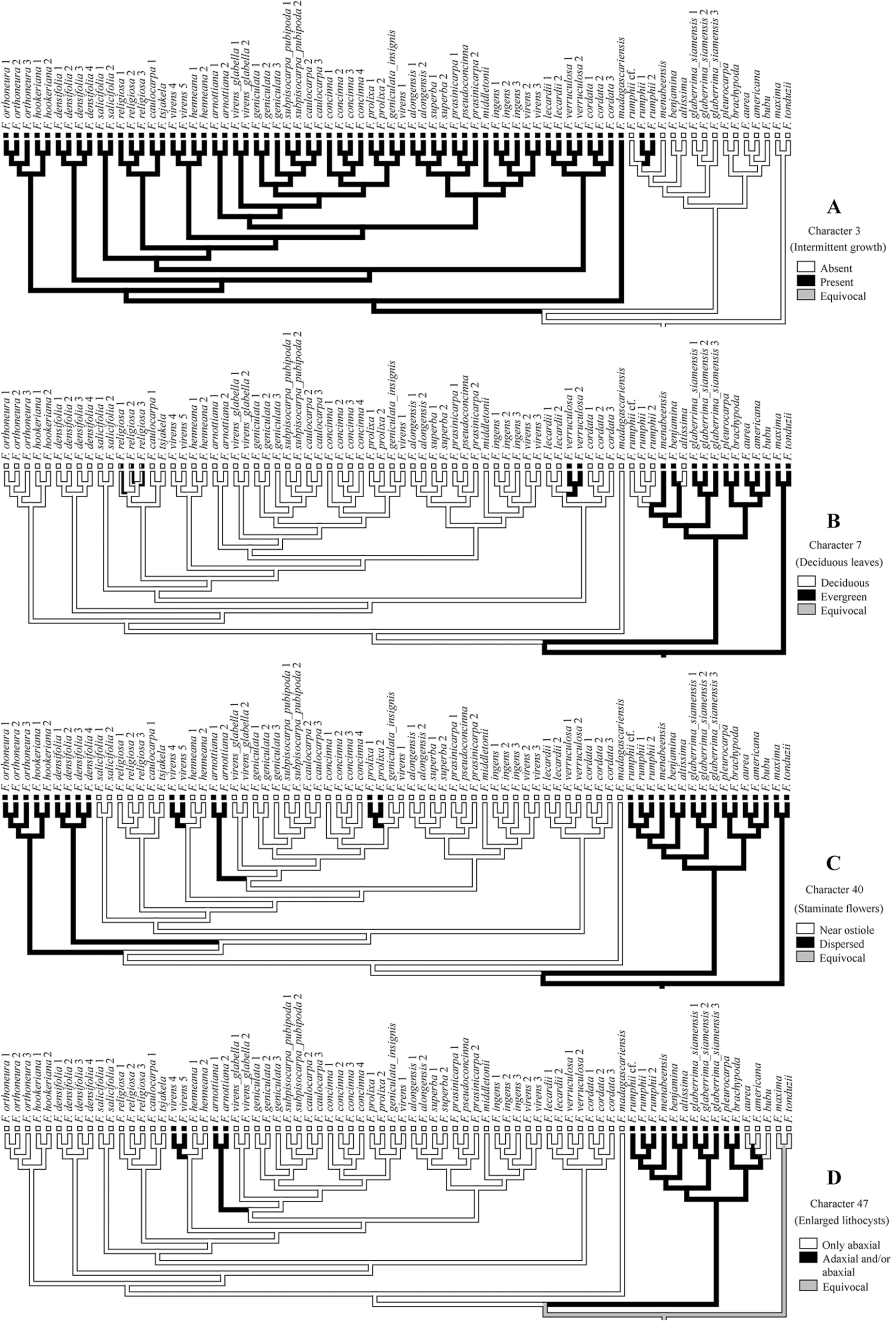
Evolution of some selected morphological and leaf anatomical characters optimized onto the phylogeny tree (Fig 2) using Mesquite v.2.7.5 with parsimony. A: Intermittent growth (character 3), B: Deciduous leaves (character 7), C: Staminate flowers (character 40), and D: Enlarged lithocysts (character 47).

Intermittent growth (char. 3, Fig 4A) was always the main character used to recognise subsect. *Urostigma*, but also occurs in parallel in *F*. *amplissima* and *F*. *rumphii*(subsect. *Conosycea*).Thus this character is homoplasious in our phylogeny and can only be used in combination with other characters to recognise subsect. *Urostigma*.

Deciduousness (char. 7, Fig 4B) is also homoplasious, and shows reversals in subsect. *Urostigma*: *F*. *verruculosa* is evergreen and *F*. *religiosa* are becomes evergreen when growing in wet areas. Moreover, several species of subsect. *Conosycea* are also deciduous. This character can respond to climatic conditions, either through phenotypic plasticity or through adaptive response over evolutionary time.

The character staminate flowers around the ostiole (char. 40, Fig 4C), the only typical morphological character, shows parallel reversals in *F*. *arnottiana*, *F*. *hookeriana*, *F*. *orthoneura*, *F*. *prolixa*, and *F*. *virens 4* and *5*. The character was used to recognise the subsection by different authors [4,5,7]. However, it may be that the character dispersed staminate flowers has evolved repeatedly within subsect. *Urostigma* in response to shifts from active to passive pollination.

Of the leaf anatomical characters, Corner [12] and Berg and Corner[7] used the enlarged lithocysts only on the abaxial surface (chr. 47, Fig 4D) as typical for subsect. *Urostigma*. However, the leaf anatomical work of Chantarasuwan et al.[16] revealed that *F*. *arnottiana* and *F*. *virens 4* and *5* show enlarged lithocysts on both sides, which is similar to subsect. *Conosycea*. Thus, this character also is not unique for subsection *Urostigma*.

The articulation of the leaf(char. 4) only occurs in Asian and Australian species, for which it is a unique apomorphy within the *Urostigma* clade, but again there are reversals to absence in *F*. *hookeriana* and *F*. *orthoneura* (perhaps related to their non-deciduousness).

### Circumscription of subsect. *Urostigma* and subsect. *Conosycea*

*Ficus amplissima* and *F*. *rumphii*, both forming former sect. *Leucogyne*, share several characters [5]: the lithocysts at both sides of the leaf blade, the dispersed staminate flowers, and whitish ovaries, while typical for subsect. *Urostigma* are the lithocysts at the abaxial side only, staminate flowers generally around the ostiole and red(-brown) ovaries [5]. *Leucogyne* and *Urostigma* are pollinated by different fig wasps, *Eupristina* in sect. *Leucogyne* and *Platyscapa* in subsect. *Urostigma*[6,7,34]. In our studysect. *Leucogyne* is not supported by phylogenetic evidence; both species areincluded in subsect. *Conosycea*. The name *Leucogyne* will then become a synonym of subsect. *Conosycea*.

Because of the reclassification of the species of former sect. *Leucogyne* the recognition of subsect. *Urostigma* and subsect. *Conosycea* changes compared to [4] and [7].

Typical for subsect. *Urostigma* are: deciduous plants, intermittent growth, articulated leaves usually present, petioles relatively long (more than 1/4^th^ of lamina long), leaves with enlarged lithocysts generally abaxially, staminate flowers usually near the ostiole.

Typical for subsect. *Conosycea*are: evergreen or deciduous plants, growth continuous, non-articulated leaves, petioles relatively thick and short (less than 1/4^th^ of lamina long), enlarged lithocysts present at both sides of the leaf lamina, figs more frequently sessile than pedunculate, staminate flowers dispersed.

### Non-monophyletic species within subsect. *Urostigma*

The sampled specimens of several species appear to be para- or polyphyletic in the results of our analysis:

#### *Ficus caulocarpa*

Three specimens of *F*. *caulocarpa* var. *caulocarpa* were included in this study of which *F*. *caulocarpa1* was separate in a clade with *F*. *tsjakela* with high PP support (Fig 2: PP = 1, BS = 79). The three specimens share many morphological characters, but *F*. *caulocarpa 1* deviates in a few characters from *F*. *caulocarpa2* and *F*. *caulocarpa3* such as the stipule forming an ovoid terminal bud, the figs present on short spurs on the branches only, and the figs solitary or in pairs. Based on these differences *F*. *caulocarpa 1* is described here as a separate species, *F*. *pseudocaulocarpa* (see below). However, in our phylogenetic analysis, the full genetic variation within *F*. *caulocarpa* is still not covered, because only samples with a narrow leaf form could be included.

#### *Ficus geniculate*

Four specimens of *F*. *geniculata* were analysed, three belong to *F*. *geniculata* var. *geniculata* and one to *F*. *geniculata* var. *insignis*. The three samples of var. *geniculata* are in different clades (paraphyletic), but var. *insignis* groups separately with *F*. *virens 1*, but with low support (Fig 2: PP = 0.8, BS = 52). Both varieties can be recognised at the species level, but because the support for the clades was low we refrain to make this decision until more molecular information becomes available.

#### *Ficus geniculata* var. *geniculate*

The two samples of *F*. *geniculata* var. *geniculata* (*1 & 2*) form a clade but with low support (Fig 2: PP = 0.8, BS = 68), while the other one (*F*. *geniculata 3*) forms a clade with *F*. *caulocarpa* and *F*. *subpisocarpa* subsp. *pubipoda*, also with low support (Fig 2: PP = 0.6, BS < 50). Because of the low support at the internal nodes, we refrain from changing the species concepts until more molecular information will be present.

#### *Ficus prasinicarpa*

The sample of *F*. *prasinicarpa 1* forms a well-supported clade with *F*. *pseudoconcinna* (Fig 2: PP = 1, BS = 87). The two are sister to *F*. *prasinicarpa 2*, but with low support. Morphologically, the two specimens of *F*. *prasinicarpa* show a difference in the leaf apex (caudate versus acute to acuminate), but because of the low support for the clade we do not make any decision about possible cryptic species.

#### *Ficus virens*

Chantarasuwan et al.[5] recognised four varieties within the *F*. *virens* complex, var. *virens*, var. *glabella*, var. *matthewii*, and var. *dispersa*. Unfortunately, we only succeeded to amplify DNA sequences from two varieties (var. *virens* and var. *glabella*). Both varieties are separated in the resulting cladogram (Fig 2), and the five samples of var. *virens*are even polyphyletic. The clade of *F*. *virens* var. *glabella* has maximum support and its morphological circumscription is clear. Therefore, we will reinstate this taxon at the species level. We will maintain *F*. *virens* with three varieties, var. *virens*, var. *dispersa*, and var. *matthewii*. *Ficus virens* var. *virens* was represented by five samples in our analyses, which became divided into three groups (Figs 1 and 2), see above. *Ficus virens 1* shows some morphological differences with *F*. *virens 2–5*, but the support is low (Fig 2: PP = 0.8, BS = 52), thus we will not change the status of*F*. *virens 1*. The morphology and leaf anatomy of the united and highly supported *F*. *virens 4* and *F*. *virens 5*(Fig 2: PP = 1, BS = 100) are distinctive from *F*. *virens 1–3*. The circumscription of *F*. *virens 4* and *5* coincides with the previous name *F*. *wightiana* (Wall. ex Miq.) Benth., which King[10] treated as *F*. *infectoria* Roxb. var. *wightiana* (Wall. ex Miq.) King, and which Corner[35] accepted as synonym of *F*. *virens*. Therefore, we will reinstate *F*. *wightiana*.

### Taxonomic Treatment

In this part we will officially make the changes in taxonomy on the basis of our phylogeny. Much of the nomenclature and descriptions can be found in Chantarasuwan et al. [5].

#### *Ficus* L. subg. *Urostigma* (Gasp.) Miq. sect. *Urostigma* (Gasp.) Endl. subsect. *Urostigma* (Gasp.) C.C. Berg

The following species can be recognized in subsection *Urostigma*:

*Ficus virens* Aiton, Hort. Kew. 3: 451. 1789—TYPE: Introduced to Kew about 1762 by James Gordon (holotype: BM).

*Ficus virens* Aiton var. *virens* Corner[5].

*Ficus virens* Aiton var. *dispersa* Chantaras. [5].

*Ficus virens* Aiton var. *matthewii* Chantaras. [5].

*Ficus glabella* Blume, Bijdr.: 452. 1825≡ *Urostigma glabellum* (Blume) Miq., Fl. Ind. Bat. 1, 2: 340. 1859≡ *Ficus virens* Aiton var. *glabella* (Blume) Corner, Gard. Bull. Singapore 17: 377. 1960—TYPE: INDONESIA. Java, Kiara beas, *Blume s*.*n*. (holotype: L; isotype: P).

*= Urostigma canaliculatum* Miq., London J. Bot. 6: 579. 1847—TYPE:AUSTRALIA. Prince of Wales Island, *Hb*. *Hooker* (holotype: K; isotype: E).

The former variety is here reinstated as species again. For more nomenclature and description see Chantarasuwan et al. ([5], under *F*. *virens* var. *glabella*).

*Ficus wightiana* (Wall. ex Miq.) Benth., Fl. Hongk.: 327. 1861≡ *Urostigma wightianum* Wall. ex Miq., London J. Bot. 6: 566. 1847 ≡ *Ficus infectoria* Roxb. var. *wightiana* (Wall. ex Miq.) King, Ann. Roy. Bot. Gard. (Culcutta) 1: 60, 63, t 75–77. 1887—TYPE: INDIA. Bangaloor, *Wallich 4540* (Herb. Wight.) (holotype: K; isotype: E).

Tree. Branches drying brown or grey-brown. Leafy twigs 3–3.5 mm thick, glabrous. Leaves with (sub)articulation; lamina elliptic, 3.8–11.0 by 2.5–5.2 cm, (sub)coriaceous, apex acuminate, the acumen sharp, base attenuate, both surfaces glabrous; lateral veins 6–10 pairs, the basal pair up to 1/5–1/3 the length of the lamina, unbranched, tertiary venation reticulate, partly parallel to lateral veins; petiole 2.0–6.5 cm long, glabrous, epidermis persistent; stipules 0.4–1.7 cm long, glabrous, persistent at the shoot apex, forming a terminal bud. Figs axillary or below the leaves, solitary or in pairs, sessile, basal bracts 1.5–3 mm long, glabrous, persistent; receptacle subglobose, 0.9–1.1 cm diam. when dry, glabrous, apex convex; ostiole 1–1.5 mm in diam., the upper ostiolar bracts glabrous; internal hairs absent. Staminate flowers dispersed, mostly pedicellate; tepals 2–3, reddish brown; stamen one. Pistillate flowers sessile or pedicellate; tepals 2–3, lanceolate or ovate, free or connate, reddish brown; ovary white to pale brown.

Note: Some samples of this species are very similar to *F*. *amplissima*. Distinctive are the elliptic leaves with an attenuate base and acuminate apex with sharp acumen.The samples *Gamble 16452* (K), *Preyadarsaman 5*(L), and *Worthington 4350*(K) were misidentified as *F*. *amplissima* by Chantarasuwan et al.[5].

*Ficus pseudocaulocarpa* Chantaras., sp. nov. [urn:lsid:ipni.org:names:77145129–1]—TYPE: PHILIPPINES, Palawan, Tatay municipality, Lake Manguao(Danao), 5 April 1984, *C*.*E*. *Ridsdale SMHI 323* (holotype: L)

Resembling *Ficus caulocarpa* (Miq.) Miq. Lamina elliptic-ovate to oblong, 3.8–11.8 by 1.8–5.2 cm, subcoriaceous; stipules 0.7–1.1 cm long, puberulous, persistent at the shoot apex, forming an ovoid terminal bud. Figs on short spurs on the older wood, solitary or in pairs.

**Fig 5:**
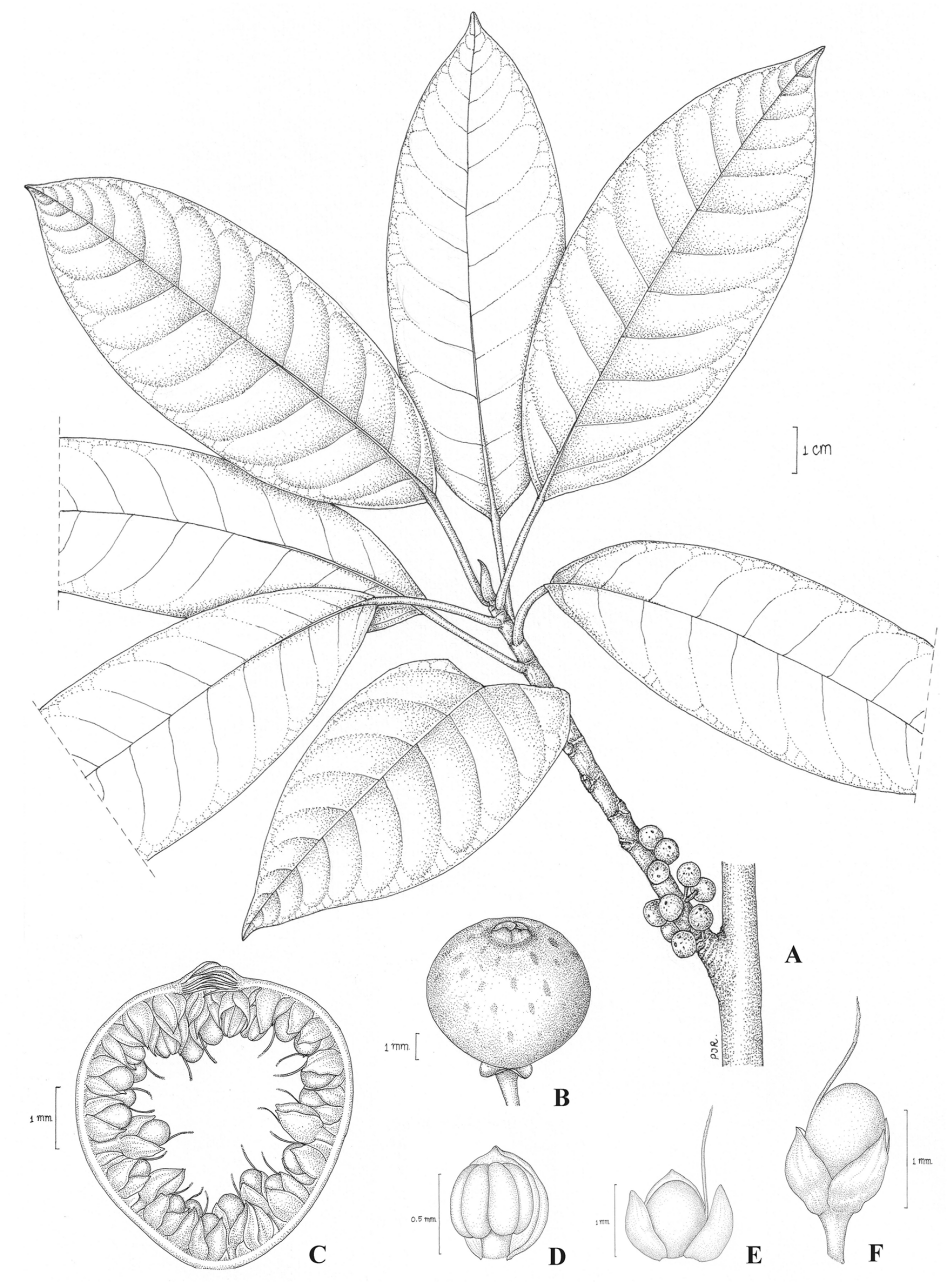
*Ficus pseudocaulocarpa* Chantaras. A: Twig with leaves and figs. B: Fig. C: Fig in longitudinal section. D: Staminate flower. E and F: Pistillate flowers.-Drawing: Pajaree Inthachup, 2014.

Tree. Branches drying brown or grey-brown. Leafy twigs3–6 mm thick, puberulous. Leaves with articulation; lamina elliptic-ovate to oblong, 3.8–11.8 by 1.8–5.2 cm, subcoriaceous, apex acute to subacuminate, the acumen blunt, base cuneate, both surfaces glabrous; lateral veins 12–16 pairs, the basal pair up to 1/6–1/4 the length of the lamina, unbranched, tertiary venation reticulate, partly parallel to lateral veins; petiole 1.3–4.5 cm long, puberulous at base, epidermis flaking off; stipules 0.7–1.1 cm long, puberulous, persistent at the shoot apex, forming an ovoid terminal bud. Figs on short spurs on the older wood, solitaryor in pairs, peduncle 0.1–0.2 cm long, glabrous or puberulous, basal bracts 1–1.5 mm long, glabrous or puberulous, persistent; receptacle subglobose, 0.4–0.5 cm diam. when dry, glabrous, apex convex; ostiole 1–1.5 mm in diam., the upper ostiolar bracts glabrous; internal hairs present. Staminate flowers near ostiole, sessile; tepals connate, reddish brown; stamen one. Pistillate flowers sessile or pedicellate; tepals 3–4, lanceolate or ovate, free or connate, reddish brown; ovary dark red.(Fig 5).

Distribution and Habitat: Philippines. In lowland rain forest at altitude 60–80 m.

Other species in this subsection see Chantarasuwan et al. [5,36]:

*Ficus alongensis* Gagnep. [5]

*Ficus arnottiana* (Miq.) Miq. [5]

*Ficus caulocarpa* (Miq.) Miq. [5]

*Ficus caulocarpa* var. *caulocarpa*[5]

*Ficus caulocarpa* var. *dasycarpa* Corner[5]

*Ficus chiangraiensis* Chantaras.[5]

*Ficus concinna* (Miq.) Miq. [5]

*Ficus cordata* Thunb. [5]

*Ficus cornelisiana* Chantaras. & Y.Q. Peng[36]

*Ficus cupulata* Haines[5]

*Ficus densifolia* Miq.[5]

*Ficus geniculata* Kurz[5]

*Ficus geniculata* var. *geniculata*[5]

*Ficus geniculata* var. *insignis* (Kurz) C.C.Berg[5]

*Ficus henneana* Miq. [5]

*Ficus hookeriana* Corner[5]

*Ficus ingens* (Miq.) Miq. [5]

*Ficus lecardii* Warb.[5]

*Ficus madagascariensis* C.C.Berg[5]

*Ficus middletonii* Chantaras.[5]

*Ficus orthoneura* H.Lév. & Vaniot[5]

*Ficus prasinicarpa* Elmer ex C.C.Berg[5]

*Ficus prolixa* G.Forst.[5]

*Ficus pseudoconcinna* Chantaras.[5]

*Ficus religiosa* L.[5]

*Ficus salicifolia* Vahl[5]

*Ficus saxophila* Blume[5]

*Ficus saxophila* subsp. *saxophila*[5]

*Ficus saxophila* subsp. *cardiophylla* (Merr.) C.C.Berg [5]

*Ficus subpisocarpa* Gagnep. [5]

*Ficus subpisocarpa* subsp. *subpisocarpa*[5]

*Ficus subpisocarpa* subsp. *pubipoda* C.C.Berg[5]

*Ficus superba* (Miq.) Miq. [5]

*Ficus tjakela* Burm.f.[5]

*Ficus verruculosa* Warb.[5]

#### *Ficus* L. subg. *Urostigma* (Gasp.) Miq. sect. *Urostigma* (Gasp.) Endl. subsect. *Conosycea* (Miq) C.C. Berg

Blumea 49: 465. 2004 ≡ *Ficus* L. subg. *Urostigma* (Gasp.) Miq. sect. *Conosycea* (Miq.) Corner,Gard. Bull. Singapore 17: 371. 1960≡ *Urostigma* Gasp. subg. *Conosycea* Miq., Fl. Ind. Bat. 1,2: 349. 1859—LECTOTYPE (designated by Corner, 1959):*Ficus annulata* Blume.

*= Urostigma* Gasp. sect. *Valida* Miq., Fl. Ind. Bat. 1,2: 334. 1859 ≡ *Ficus* L. subg. *Urostigma* (Gasp.) Miq. ser. *Validae*(Miq.) Miq., Ann. Mus. Bot. Lugduno–Batavi 3: 285. 1867; Corner, Gard. Bull. Singapore 17: 272. 1960—LECTOTYPE (designated by Corner, 1959):*Urostigma valida* (Blume) Miq. [= *Ficus annulata* Blume].

*= Ficus* L. sect. *Stilpnophyllum* Endl. subsect. *Sessiliflorae* Sata, Contr. Hort. Inst. Taihoku Imp. Univ. 32: 179, 190, 375, 376. 1944—TYPE: unknown.

*= Ficus* L. subg. *Urostigma* (Gasp.) Miq. sect. *Conosycea* (Miq.) Corner subsect. *Conosycea* (Miq.) C.C. Berg ser. *Drupaceae* Corner, Gard. Bull. Singpore 17: 372. 1960 ≡ *Ficus* L. subg. *Urostigma* (Gasp.) Miq. sect. *Conosycea* (Miq.) Corner ser. *Drupaceae* Corner subser. *Drupaceae* Corner, Gard. Bull. Singapore 17:372. 1960—TYPE:*Ficus drupacea* Thunb.

*= Ficus* L. subg. *Urostigma* (Gasp.) Miq. sect. *Conosycea* (Miq.) Corner subsect. *Conosycea* (Miq.) C.C. Berg ser. *Drupaceae* Corner subser. *Indicae* Corner, Gard. Bull. Singapore 17: 372. 1960 ≡ *Perula* Raf., Sylv. Tellur.: 59.1838, non Schreb. 1791—TYPE:*Ficus benghalensis* L.

*= Ficus* L. subg. *Urostigma* (Gasp.) Miq. sect. *Conosycea* (Miq.) Corner subsect. *Conosycea* (Miq.) C.C. Berg ser. *Drupaceae* Corner subser. *Zygotricheae* Corner, Gard. Bull. Singapore 17: 372. 1960—TYPE:*Ficus consociata* Blume

*= Ficus* L. subg. *Urostigma* (Gasp.) Miq. sect. *Conosycea* (Miq.) Corner subsect. *Conosycea* (Miq.) C.C. Berg ser. *Drupaceae* Corner subser. *Crassirameae* Corner, Gard. Bull. Singapore 17: 373. 1960—TYPE:*Ficus crassiramea* Miq.

*= Ficus* L. subg. *Urostigma* (Gasp.) Miq. sect. *Conosycea* (Miq.) Corner subsect. *Dictyoneuron* Corner, Gard. Bull. Singapore 17: 373. 1960—TYPE:*Ficus sundaica* Blume

*= Ficus* L. subg. *Urostigma* (Gasp.) Miq. sect. *Conosycea* (Miq.) Corner subsect. *Dictyoneuron* Corner ser. *Dubiae* Corner, Gard. Bull. Singapore 17: 373. 1960—TYPE:*Ficus dubia* Wall. ex King

*= Ficus* L. subg. *Urostigma* (Gasp.) Miq. sect. *Conosycea* (Miq.) Corner subsect. *Dictyoneuron* Corner ser. *Glaberrimae* Corner, Gard. Bull. Singapore 17: 373. 1960—TYPE:*Ficus glaberrima* Blume

*= Ficus* L. subg. *Urostigma* (Gasp.) Miq. sect. *Conosycea* (Miq.) Corner subsect. *Dictyoneuron* Corner ser. *Subvalidae* (Miq.) Corner, Gard. Bull. Singapore 17: 373. 1960 ≡ *Urostigma* Gasp. sect. *Subvalida* Miq., Fl. Ind. Bat. 1,2: 339. 1859—TYPE:*Ficus sundaica* Blume

*= Ficus* L. subg. *Urostigma* (Gasp.) Miq. sect. *Conosycea* (Miq.) Corner subsect. *Dictyoneuron* Corner ser. *Perforatae* Corner, Gard. Bull. Singapore 17: 374. 1960—TYPE:*Ficus pisocarpa* Blume

*= Ficus* L. subg. *Urostigma* (Gasp.) Miq. sect. *Conosycea* (Miq.) Corner subsect. *Benjamina* (Miq.) Corner, Gard. Bull. Singapore 17: 374. 1960 ≡ *Ficus* L. subg. *Urostigma* (Gasp.) Miq. ser. *Benjamineae* Miq. Ann. Mus. Bot. Lugduno–Batavi 3: 287. 1867—TYPE:*Ficus benjamina* L.

*= Ficus* L. subg. *Urostigma* (Gasp.) Miq. sect. *Conosycea* (Miq.) Corner subsect. *Benjamina* (Miq.) Corner ser. *Callophylleae* Corner, Gard. Bull. Singapore 17: 374. 1960—TYPE:*Ficus callophylla* Blume

*= Ficus* L. subg. *Urostigma* (Gasp.) Miq. sect. *Leucogyne* Corner, Gard. Bull. Singapore 17: 371. 1960—TYPE: *Ficus rumphii* Blume

Trees, mostly evergreen, without intermittent growth to rarely intermittent growth with 2 or 3 short internodes forming a transition zone. Leaves spirally arranged, not articulate; epidermis multiple, enlarged lithocysts at both sides of lamina; petiole relatively thick and short. Figs solitary or in pairs axillary, or just below the leaves, more frequently sessile than pedunculate; receptacle often longer than wide; basal bracts 3(2), small to large, often unequal in size or shape, mostly persistent; ostiole closed, with the upper ostiolar bracts overlapping, or open, with the upper ostiolarbracts not or partly imbricate, the 3 upper ostiolar bracts often unequal in size, sometimes only 2 clearly visible; internal hairs mostly absent; staminate flowers dispersed; tepals mostly red(dish) brown; ovary mostly white or partly reddish, sometimes entirely reddish.

*Ficus amplissima* J.E.Sm. in Rees, Cycl. 14: n. 68. 1810, non Miq. 1867; Corner, Gard. Bull. Singapore 18: 84. 1961; 21: 11. 1965; K.M.Matthew, Fl. Tam. Carnatic 3: 1515. 1983 ≡ *Tsjela* Rheede, Hort. Mal. 3: 85, t. 63. 1682, nom. inval. ≡ *Ficus tsiela* Roxb, Hort. Bengal.: 66. 1826, nom. superfl.; Fl. Ind. 3: 549. 1832; King in Hook.f., Fl. Brit. India 5: 515. 1888. ≡ *Ficus tsjela* Roxb. ex Buch.–Ham., Tr. Linn. Soc. 15: 149. 1826, nom. superfl.; King, Ann. Roy. Bot. Gard. (Culcutta) 1: t.74. 1887 ≡ *Ficus indica* auct. non L.: L., Sp. Pl. 2: 1060. 1753; Vahl. Enum. Pl., ed. 2: 195. 1806; Willd., Sp. Pl., ed. 4, 4(2): 1146. 1806.–TYPE: Rheede (1682) t. 63, based on *Tsjela* Rheed.

= *Urostigma pseudobenjamineum* Miq., London J. Bot. 6: 566. 1847 ≡ *Ficus pseudobenjaminea* (Miq.) Miq., Ann. Mus. Bot. Lugduno–Batavi 3: 286. 1867 –TYPE: INDIA. Luddaloor, *Wight s*.*n*. in Herb. Rupel (holotype: K).

= *Urostigma pseudotsiela* Miq., London J. Bot. 6: 566. 1847. ≡ *Ficus pseudotsiela* (Miq.) King, Ann. Roy. Bot. Gard. (Culcutta) 1: t. 74. 1887 –TYPE: *Wight*. in Herb. Hook. (not found yet, information based on Corner 1965).

*Ficus rumphii* Blume, Bijdr. Fl. Ned. Ind. 9: 437. 1825; Miq., Ann. Mus. Bot. Lugduno–Batavi 3: 287. 1867; King, Ann. Roy. Bot. Gard. (Calcutta) 1: 54, t. 67B. 1887; Gagnep.in Lecomte, Fl. Indo–Chine 5: 768. 1928; Corner, Wayside Trees 1: 687. 1940; Gard.Bull. Singapore 21: 11. 1965; C.C.Berg and Corner in Nooteb., Fl. Males. Ser. 1, 17 (2): 609.2005 ≡ *Urostigma rumphii* (Blume) Miq. in Zoll., Syst. Verz. 2: 90. 1854; Fl. Ind. Bat. 1,2: 322. 1859—TYPE: INDONESIA. Java, *Reinwardt 1121* (holotype: L; isotype: P).

*=* [*Ficus populiformis* Schott ex Miq., Ann. Mus. Bot. Lugduno–Batavi 3: 287. 1867, nom.nud.]

*= Ficus religiosa* L. var. ß “*Arbor conciliorum* etc.” Lam., Encycl. 2, 2: 493. 1788. nom. illig.–*Ficus cordifolia* Roxb., Fl. Ind. (Carey ed.) 3: 548. 1832 ≡ *Urostigma cordifolium*(Roxb.) Miq., London J. Bot. 6: 564. 1847 ≡ *Ficus conciliorum* Oken, Allg. Naturgesch.3: 1561. 1841, nom. superfl.—TYPE: based on Rumphius: *Arbor conciliorum* Rumph.,Herb. Amboin. 3: t.91, 92. 1743.

*= Ficus damit* Gagnep., Notul. Syst. (Paris) 4: 88. 1927; in Lecomte, Fl. Indo–Chine 5: 812, f.93. 1928—TYPE: VIETNAM. Quang–tri, Lao–bao, *Poilane 1337* (holotype: P).

*Ficus pubipetiola* Chantaras., sp. nov. [urn:lsid:ipni.org:names:77147190–1]—TYPE: THAILAND, Lop Buri, Tha Wung, Wat Khao Samorkhorn, 18 September 2010, *Chantarasuwan 180910–2*, (holotype: THNHM, isotype: L).

Leaf lamina ovate, 4–9 by 6.5–12 cm, subcoriaceous, apex (sub)acuminate, pubescent on midrib and primary veins on lower surface, petiole 1.1–2.5 cm long, pubescent. Figs axillary, sessile.

**Fig 6:**
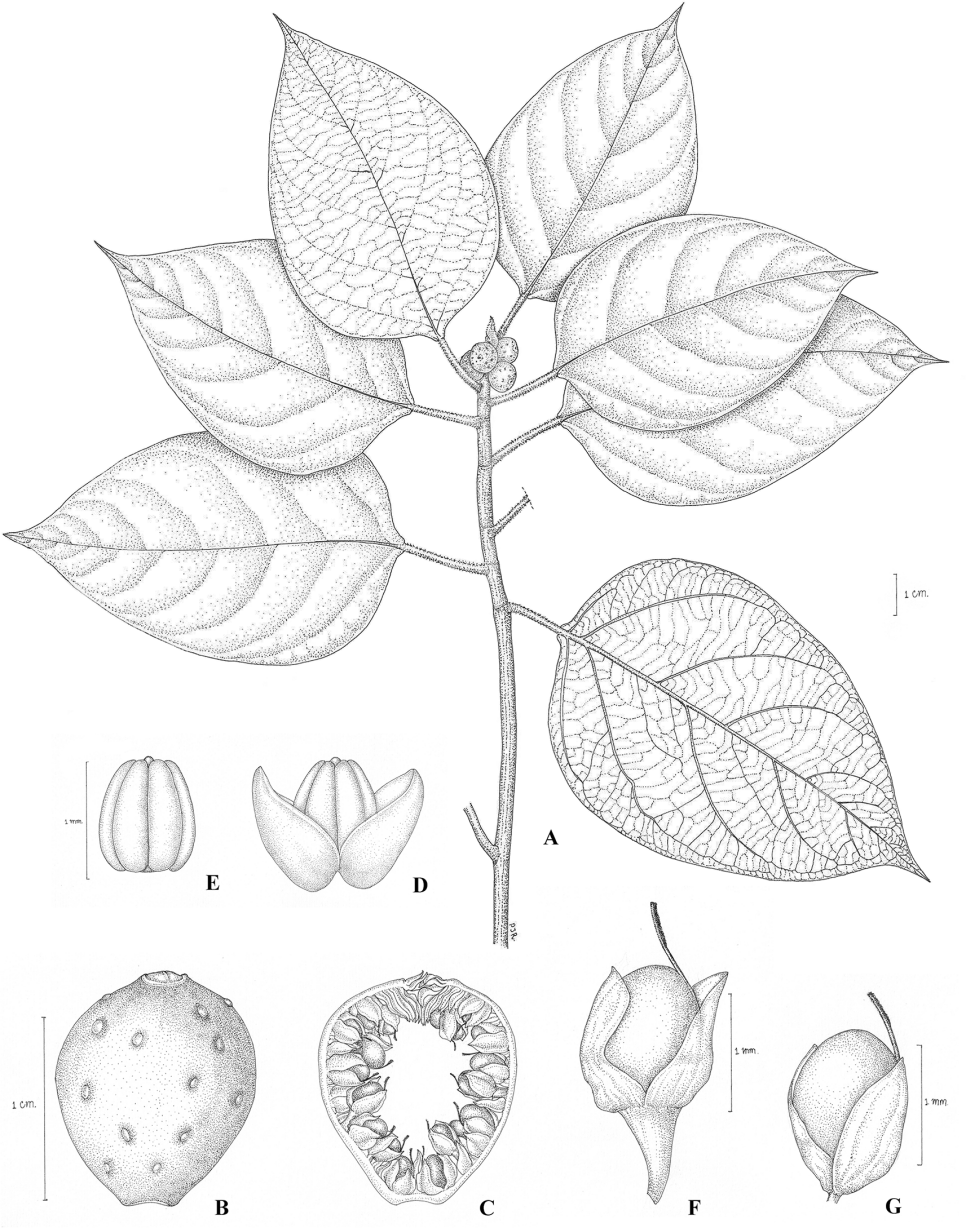
*Ficus pubipetiola* Chantaras. A: Twig with leaves and figs. B: Fig. C: Fig in longitudinal section. D: Staminate flower. E: Anther. F and G: Pistillate flowers.-Drawing: Pajaree Inthachup, 2014.

Small trees, up to 7 m tall, branches drying grey-brown, without intermittent growth. Leafy twigs 2–4 mm thick, pubescent, epidermis flaking off. Leaves spirally arranged, not articulate; lamina ovate, 4–9 by 6.5–12 cm, subcoriaceous, apex (sub)acuminate, the acumen sharp, base broadly cuneate or sub-attenuate, rarely sub-cordate, upper surface glabrous except pubescent on midrib, lower surface glabrous except pubescent on midrib and primary veins; lateral veins 5–9 pairs, furcated away from margin, the basal pair up to ¼–2/5^th^ the length of the lamina, branched, tertiary venation reticulate; petiole 1.1–2.5 cm long, pubescent, epidermis persistent. Stipules 0.8–1.7 cm long, brown pubescent, persistent at tip of twig. Figs axillary, solitary or in pairs, sessile; basal bracts 3, 1–2 mm long, glabrous, persistent, receptacle obovate, 0.8–1.1 cm in diam. when dry, glabrous, apex convex, ostiole 2–2.5 mm in diam., upper ostiolar bractsglabrous; internal hairs absent. Staminate flowers dispersed, sessile to pedicellate;tepals 3, ovate to broad-lanceolate, free, red-brown; stamen one. Pistillate flowers sessile to pedicellate, sometimes with a bract at base of pedicel; tepals 3, ovate or broadly lanceolate, free, red–brown; ovary white (or pale yellow).(Fig 6).

Distribution and Habitat: Thailand, on limestone in dwarf community, at elevation of c. 30 m.Figsin September–November.

Other species in this subsection are:

*Ficus acamptophylla* (Miq.) Miq. [7]

*Ficus altissima* Blume[7]

*Ficus annulata* Blume[7]

*Ficus archboldiana* Summerh.[7]

*Ficus balete* Merr.[7]

*Ficus benghalensis* L.[7]

*Ficus benjamina* L.[7]

*Ficus binnendijkii* (Miq.) Miq.[7]

*Ficus borneensis* Kochummen[7]

*Ficus bracteata* (Wall. ex Miq.) Miq.[7]

*Ficus callophylla* Blume[7]

*Ficus chrysolepis* Miq.[7]

*Ficus chrysolepis* subsp. *chrysolepis*[7]

*Ficus chrysolepis* subsp. *novoguineensis* (Corner) C.C. Berg[7]

*Ficus consociata* Blume[7]

*Ficus cordatula* Merr.[7]

*Ficus corneri* Kochummen[7]

*Ficus costata* Aiton[36]

*Ficus crassiramea* (Miq.) Miq.[7]

*Ficus crassiramea* subsp. *crassiramea*[7]

*Ficus crassiramea* subsp. *stupenda* (Miq.) C.C. Berg[7]

*Ficus cucurbitina* King[7]

*Ficus curtipes* Corner[7]

*Ficus dalhousiae* Miq.[32]

*Ficus delosyce* Corner[7]

*Ficus depressa* Blume[7]

*Ficus drupacea* Thunb.[7]

*Ficus dubia* Wall. ex King[7]

*Ficus fergusoni* (King) Worthington[35]

*Ficus forstenii* Miq.[7]

*Ficus glaberrima* Blumea[37]

*Ficus glaberrima* subsp. *glaberrima*[37]

*Ficus glaberrima* subsp. *siamensis* (Corner) C.C. Berg[37]

*Ficus globosa* Blume[7]

*Ficus humbertii* C.C. Berg [6]

*Ficus involucrata* Blume[7]

*Ficus juglandiformis* King[7]

*Ficus kerkhovenii* Valeton[7]

*Ficus kochummeniana* C.C. Berg[7]

*Ficus kurzii* King[7]

*Ficus lawesii* King[7]

*Ficus lowii* King[7]

*Ficus maclellendii* King[7]

*Ficus menabeensis* Perrier [6]

*Ficus microcarpa* L.f.[7]

*Ficus microsyce* Ridl.[7]

*Ficus miqueliana* C.C. Berg[7]

*Ficus mollis* Vahl[35]

*Ficus pallescens* (Weiblen) C.C. Berg[7]

*Ficus paracamptophylla* Corner[7]

*Ficus patellata* Corner[7]

*Ficus pellucidopunctata* Griff.[7]

*Ficus pisocarpa* Blume[7]

*Ficus pubilimba* Merr.[7]

*Ficus retusa* L. [7]

*Ficus rigo* F.M.Bailey[7]

*Ficus soepadmoi* Kochummen[7]

*Ficus spathulifolia* Corner[7]

*Ficus stricta* (Miq.) Miq.[7]

*Ficus subcordata* Blume[7]

*Ficus subgelderi* Corner[7]

*Ficus sumatrana* (Miq.) Miq.[7]

*Ficus sundaica* Blume[7]

*Ficus talbotii* King (= *F*. *calcicola* Corner) [37]

*Ficus tristaniifolia* Corner[7]

*Ficus xylophylla* (Wall. ex Miq.) Miq.[7]

## Supporting Information

S1 Appendix Species, voucher specimen, and Gen Bank information for sequence data reported in the study: sequence per entry: Species; Taxon code; Voucher; Source and Geographic regions; GenBank accession(ITS, ETS, G3pdh, ncpGS). (DOCX)

S2 Appendix List of morphological and leaf anatomical characters used in the phylogenetic analysis. Characters 8, 9, 11, 12, 13, 18, 21, 33, 34, 37, 45, and 63 are continuously distributed. However, these characters are coded as having discrete states, because several characters show a gap (9, 18, 21, 45) or a soft gap, whereby the few taxa with overlap are coded as polymorphic (both states present).
(DOCX)

S3 Appendix Data matrix of morphological(1–43) and leaf anatomical(44–66) characters scored for the phylogenetic analyses and characterreconstruction. Polymorphisms are indicated by all states presented by a comma, and inapplicable or unknown characters by “-”. etails of characters and states are also listed below.
(DOCX)
