# Supplementary material for: A New Classification of Ficus Subsection Urostigma (Moraceae) Based on Four Nuclear DNA Markers (ITS, ETS, G3pdh, and ncpGS), Morphology and Leaf Anatomy
Source: PLoS One. 2015 Jun 24;10(6):e0128289. doi: 10.1371/journal.pone.0128289 (PMC4479584; doi:10.1371/journal.pone.0128289)
Supplement: S1 Appendix — (DOCX) [file pone.0128289.s001.docx]

**S1 APPENDIX. Species, voucher specimen, and Gen Bank information for sequence data reported in the study**: sequence per entry: Species; Taxon code;Voucher; Source and Geographic regions; GenBank accession(ITS, ETS, G3pdh, ncpGS)

*Ficus alongensis* Gagnep.; *alongensis*1; Steward and Cheo 1187(P); China, Shaanxi, Chang An; KJ845962, KJ845902, KJ846015, -

*Ficus alongensis* Gagnep.; *alongensis*2; R.C. Ching 1917(P); China; KJ845963, KJ845903, - , -

*Ficus arnottiana* (Miq.) Miq.; *arnottiana* 1; A.H.M. Jayasuriya 1293 (L); Sri Lanka, Anuradhapura, Ritigala Strict Natural reserve; - , KJ845879, - , -

*Ficus arnottiana* (Miq.) Miq.; *arnottiana* 2; 2038(no collector name) (L); India, Mangalor; - , KJ845880, - , -

*Ficus caulocarpa* (Miq.) Miq.; *caulocarpa*1; C.E. Ridsdale SMHI 323 (L); Philippines, Taytay municipality, Lake Manguao; KJ845953, - , - , -

*Ficus caulocarpa* (Miq.) Miq.; *caulocarpa*2; Chantarasuwan 261111-1(L); Thailand, Trang, Nayong; KJ845954, KJ845894, KJ846009, -

*Ficus caulocarpa* (Miq.) Miq.; *caulocarpa*3; Chantarasuwan 071010-2 (L); Thailand, Nakhon Si Thammarat, Noppitam; KJ845955, KJ845895, KJ846010, -

*Ficus concinna* (Miq.) Miq.; *concinna*1; Chantarasuwan 071010-1 (L); Thailand, Nakhon Si Thammarat, Thasala; KJ845989, KJ845928, KJ846035, -

*Ficus concinna* (Miq.) Miq.; *concinna*2; Chantarasuwan 140910-3 (L); Thailand, Ratchaburi, Chombung; KJ845990, KJ845929, KJ846036, -

*Ficus concinna* (Miq.) Miq.; *concinna*3; Chantarasuwan 120910-5(L); Thailand, Rayong, Pe; KJ845991, KJ845930, KJ846037, KJ846071

*Ficus concinna* (Miq.) Miq.; *concinna*4; Chantarasuwan 051010-4(L); Thailand, PrachuapKhiri Khan, Kuiburi; KJ845992, KJ845931, KJ846038, KJ846072

*Ficus cordata* Thunb.; *cordata*1; Dinter 275 (WAG); Namibia; KJ845973, KJ845912, KJ846020, -

*Ficus cordata* Thunb.; *cordata*2; Seydel 1555 (WAG); Namibia, Erongo, Okongawa; KJ845974, 845913, KJ846021, -

*Ficus cordata* Thunb.; *cordata*3; Theson 3363 (WAG); Namibia; KJ845975, KJ845914, KJ846022, KJ846063

*Ficus densifolia* Miq.; *densifolia*1; Baider CB2421 (L); Mauritius; KJ845983, KJ845922, KJ846030, KJ846068

*Ficus densifolia* Miq.; *densifolia*2; Baider CB2422 (L); Mauritius; KJ845984, KJ845923, KJ846031, KJ846069

*Ficus densifolia* Miq.; *densifolia*3; M02 (CEFE-CNRS); Mauritius; KJ845985, KJ845924, KJ846032, -

*Ficus densifolia* Miq.; *densifolia*4; M01 (CEFE-CNRS); Mauritius; KJ845986, KJ845925, KJ846033, KJ846070

*Ficus geniculata* Kurz var. *geniculata*; *geniculata*1; Chantarasuwan 150910-1 (L); Thailand, Kanchanaburi, Thong PhaPhum, Lintin; KJ845940, KJ845882, KJ845999, KJ846044

*Ficus geniculata* Kurz var. *geniculata*; *geniculata*2; Chantarasuwan 210910-1 (L); Thailand, Lamphun, Muang; KJ845941, KJ845883, KJ846000, KJ846045

*Ficus geniculata* Kurz var. *geniculata*; *geniculata*3; Chantarasuwan 301111-1 (L); Thailand, Chiang Rai, Muang, Pongsali; KJ845942, KJ845884, - , KJ846046

*Ficus geniculata* Kurz var. *insignis* (Kurz) C.C.Berg; *geniculate-insignis*; Parker 1144 (L); Australia, Northern Territory, Darwin; KJ845943, KJ845885, KJ846001, KJ846047

*Ficus glaberrima* Blume subsp. *siamensis* (Corner) C.C.Berg; *glaberrima*-*siamensis*1; Chantarasuwan 110910-2 (L); Thailand, Sa Kaeo, KhaoChakan; KJ845996, KJ845935, KJ846041, KJ846076

*Ficus glaberrima* Blume subsp. *siamensis* (Corner) C.C.Berg; *glaberrima*-*siamensis*2; Chantarasuwan 110910-3 (L); Thailand, Sa Kaeo, KhaoChakan; KJ845997, KJ845936, KJ846042, KJ846077

*Ficus glaberrima* Blume subsp. *siamensis* (Corner) C.C.Berg; *glaberrima*-*siamensis*3; Chantarasuwan 180910-3 (L); Thailand, Lop Buri, Thawung; KJ845998, KJ845937, KJ846043, -

*Ficus henneana* Miq.; *henneana*1; J.R. Maconochie 2208 (L); Australia, Arnhem Land, Elcho Isl.; KJ845967, - , KJ846016, KJ846058

*Ficus henneana* Miq.; *henneana*2; B. Hyland 8086 (L); Australia, Queensland, Atherton; KJ845968, KJ845907, - , KJ846059

*Ficus hookeriana* Corner; *hookeriana*; Hooker&T.Thomson 120 (L); India, Sikkim; KJ845988, KJ845927, - , -

*Ficus ingens* (Miq.) Miq.; *ingens* 1; BG 03 (L); Ivory Coast; KJ845964, KJ845904, - , KJ846056

*Ficus ingens* (Miq.) Miq.; *ingens*2; Correia 3777 (WAG); Mozambique; KJ845965, KJ845905, - , -

*Ficus ingens* (Miq.) Miq.; *ingens*3; Jongkind 4317 (WAG); Ivory Coast; KJ845966, KJ845906, - , KJ846057

*Ficus lecardii* Warb.; *lecardii*1; Harris 2136 (WAG); Central African Republic; KJ845971, KJ845910, KJ846018, KJ846061

*Ficus lecardii* Warb.; *lecardii*2; Letouzey 6949 (WAG); Cameroon; KJ845972, KJ845911, KJ846019, KJ846062

*Ficus madagascariensis* C.C.Berg; *madagascariensis*; P.R. Montagnac 72 (WAG); Madagascar, without locality; KJ845956, KJ845896, - , KJ846053

*Ficus middletonii* Chantaras.; *middletonii*; Chantarasuwan 051010-2 (L); Thailand, Prachuap Khiri Khan, Kuiburi; KJ845952, KJ845893, KJ846008, KJ846052

*Ficus orthoneura* H.Lév. &Vaniot; *orthoneura* 1; Chantarasuwan 231111-1 (L); Thailand, Tak, Phobpra; KJ845987, KJ845926, KJ846034, -

*Ficus prasinicarpa* Elmer ex C.C.Berg; *prasinicarpa*1; Ridsdale 434 (L); Philippines; KJ845947, - , - , -

*Ficus prasinicarpa* Elmer ex C.C.Berg; *prasinicarpa*2; Nagari 7309 (L); Papua New Guinea; KJ845948, KJ845889, - , -

*Ficus prolixa* G. Forst.; *prolixa*1; Gillett 2206 (L); Marquesas; KJ845949, KJ845890, KJ846005, KJ846051

*Ficus prolixa* G. Forst.; *prolixa*2; Fosberg 25302 (L); Guam; KJ845950, KJ845891, KJ846006, -

*Ficus pseudoconcinna* Chantaras.; *pseudoconcinna*; Soenarko 355 (L); Indonesia, Sulawesi; KJ845946, KJ845888, KJ846004, KJ846050

*Ficus religiosa* L.; *religiosa*1; BG 04(L); unknown; KJ845980, KJ845919, KJ846027, KJ846066

*Ficus religiosa* L.; *religiosa*2; Chantarasuwan 110910-4 (L); Thailand, Sa Kaeo, Khao Chakan; KJ845981, KJ845920, KJ846028, -

*Ficus religiosa* L.; *religiosa*3; Chantarasuwan 150910-2 (L); Thailand, Kanchanaburi, Thong Pha Phum, Lintin; KJ845982, KJ845921, KJ846029, KJ846067

*Ficus* cf. *rumphii*; *rumphii*cf.; Chantarasuwan 180910-2 (L); Thailand, Lop Buri, Thawung; KJ845995, KJ845934, - , KJ846075

*Ficus rumphii* Blume; *rumphii*1; Chantarasuwan 120910-4 (L); Thailand, Rayong, Pe; KJ845993, KJ845932, KJ846039, KJ846073

*Ficus rumphii* Blume; *rumphii*2; Chantarasuwan 140910-1 (L); Thailand, Ratchaburi, Chombung; KJ845994, KJ845933, KJ846040, KJ846074

*Ficus salicifolia* Vahl; *salicifolia*1; Humberts.n. (WAG); South Africa; KJ845976, KJ845915, KJ846023, KJ846064

*Ficus salicifolia* Vahl; *salicifolia*2; Bornmüller 646 (WAG); Saudi Arabia; KJ845977, KJ845916, KJ846024, -

*Ficus subpisocarpa* Gagnep. subsp. *pubipoda* C.C. Berg; *subpisocarpa-pubipoda*1; Chantarasuwan 110910-1 (L); Thailand, Chachoengsao, Panom Sarakham; KJ845969, KJ845908, - , -

*Ficus subpisocarpa* Gagnep. subsp. *pubipoda* C.C. Berg; *subpisocarpa-pubipoda*2; Chantarasuwan 011211-1 (L); Thailand, Chachoengsao, Panom Sarakham; KJ845970, KJ845909, KJ846017, KJ846060

*Ficus superba* (Miq.) Miq.; *superba*1; C. Friedberg 138 (L); Indonesia,Timor central; KJ845944, KJ845886, KJ846002, KJ846048

*Ficus superba* (Miq.) Miq.; *superba*2; Chantarasuwan 120910-2 (L); Thailand, Rayong, Kleang; KJ845945, KJ845887, KJ846003, KJ846049

*Ficus tsjakela* Burm.f.; *tsjakela*; Kostermans 27682 (L); Sri Lanka, Botanics Garden Peradeniya; KJ845951, KJ845892, KJ846007, -

*Ficus verruculosa* Warb.; *verruculosa*1; Radcliff-Smith 5982 (WAG); Malawi; KJ845978, KJ845917, KJ846025, -

*Ficus verruculosa* Warb.; *verruculosa*2; Adjakidje 2779 (WAG); Benin; KJ845979, KJ845917, KJ846026, KJ846065

*Ficus virens* Aiton var. *glabella* (Blume) Corner; *virens-glabella*1; Chantarasuwan 071010-3 (L); Thailand, Nakhon Si Thammarat, Noppitam; KJ845960, KJ845900, KJ846013, KJ846055

*Ficus virens* Aiton var. *glabella* (Blume) Corner; *virens-glabella*2; Chantarasuwan 071010-4 (L); Thailand, Nakhon Si Thammarat, Noppitam; KJ845961, KJ845901, KJ846014, -

*Ficus virens* Aiton var. *virens*; *virens*1; P. Martensz AE 257 (L); Australia, Northern Territory; KJ845957, KJ845897, KJ846011, KJ846054

*Ficus virens* Aiton var. *virens*; *virens*2; E. Jacobson 2191 (L); Indonesia ,Sumatra; KJ845958, KJ845898, KJ846012, -

*Ficus virens* Aiton var. *virens*; *virens*3; G. Leach UPNG 3747 (L); Papua New Guinea, Central Province; KJ845959, KJ845899, - , -

*Ficus virens* Aiton var. *virens*; *virens* 4; L.H. Cramer 4670 (L); Sri Lanka, North-Western province, Puttalam, Talawila; KJ845938, KJ845881, - , -

*Ficus virens* Aiton var. *virens*; *virens* 5; Preyadasaman 5 (L); India, Coimbatore; KJ845939, - , - , -
