# Supplementary material for: A New Classification of Ficus Subsection Urostigma (Moraceae) Based on Four Nuclear DNA Markers (ITS, ETS, G3pdh, and ncpGS), Morphology and Leaf Anatomy
Source: PLoS One. 2015 Jun 24;10(6):e0128289. doi: 10.1371/journal.pone.0128289 (PMC4479584; doi:10.1371/journal.pone.0128289)
Supplement: S2 Appendix — Characters 8, 9, 11, 12, 13, 18, 21, 33, 34, 37, 45, and 63 are continuously distributed. However, these characters are coded as having discrete states, because several characters show a gap (9, 18, 21, 45) or a soft gap, whereby the few taxa with overlap are coded as polymorphic (both states present). (DOCX) [file pone.0128289.s002.docx]

**S2 APPENDIX. List of morphological and leaf anatomical characters used in the phylogenetic analysis.** Characters 8, 9, 11, 12, 13, 18, 21, 33, 34, 37, 45, and 63 are continuously distributed. However, these characters are coded as having discrete states, because several characters show a gap (9, 18, 21, 45) or a soft gap, whereby the few taxa with overlap are coded as polymorphic (both states present).

1. Habit: (1) shrub; (2) tree. 2. Aerial roots: (1) present; (2) absent. 3. Intermittent growth: (1) present; (2) absent. 4. Leaf articulation: (1) present; (2) absent. 5. Indumentum of leafy twig: (1) glabrous to puberulous; (2) tomentose to villous. 6. Periderm of leafy twig: (1) persistent; (2) flaking off. 7. Leaf persistence: (1) deciduous; (2) evergreen. 8. Position of broadest part of leaf: (1) base; (2) middle; (3) apex. 9. Relative presence of cordate leaves: (1) cordate leaves dominant (>50%) (2) cordate leaves not dominant (<50%). 10. Caudate leaf apex: (1) present; (2) absent. 11. Relative width of lamina compared to length: (1) broad (> ¼); (2) narrow (< ¼). 12. Relative length of basal pair of nerves: (1) ≤ 1/3 of lamina; (2) > 1/3 of lamina. 13. Number of lateral veins: (1) 4-9; (2) ≥ 10. 14. Branching of lateral veins: (1) present; (2) absent. 15. Branching of basal veins: (1) present; (2) absent. 16. Tertiary venation: (1) partly parallel with primary veins; (2) reticulate. 17. Waxy glands: (1) at base of midrib; (2) in axil of lateral veins (can be regarded as two characters, but taxa do not show overlap, therefore, states considered homologous). 18. Relative petiole length: (1) < ¼ of leaf length; (2) > ¼ of leaf length. 19. Epidermis of petiole: (1) persistent; (2) flaking off. 20. Indumentum of petiole: (1) glabrous; (2) puberulous. 21. Length of stipules: (1) < 1 cm long; (2) ≥ 1 cm long. 22. Stipular bracts: (1) forming broadly ovoid terminal bud; (2) forming ovoid terminal bud. 23. Epidermis of stipule: (1) persistent; (2) flaking off. 24. Indumentum of stipule: (1) glabrous to puberulous; (2) tomentose to villous. 25. Persistence of stipule: (1) persistent; (2) caducous. 26. Ramiflorous figs: (1) with spur; (2) without spur. 27. Grouping of figs: (1) 1 or 2; (2) 3 to 8. 28. Indumentum of figs: (1) glabrous to puberulous; (2) tomentose to villous. 29. Fig peduncle: (1) present; (2) absent. 30. Number of basal bracts: (1) 2; (2) 3. 31. Persistence of basal bracts: (1) persistent; (2) caducous. 32. Degree of covering of fig by basal bracts: (1) only base of fig; (2) up to middle of fig. 33. Fig size: (1) 0.4–1 cm in diam. when dry; (2) > 1 cm in diam. when dry. 34. Fig form: (1) ovate to subglobose; (2) obovate to subpyriform; (3) oblong. 35. Colour of fig at maturity: (1) orange-red; (2) black; (3) green. 36. Apex of fig: (1) convex; (2) flat; (3) concave. 37. Size of ostiole: (1) 1–3 mm in diam.; (2) > 3 mm in diam. 38. Indumentum of ostiolar bracts: (1) glabrous; (2) puberulous. 39. Internal hairs of fig: (1) present; (2) absent. 40. Position on staminate flowers: (1) near ostiole; (2) dispersed. 41. Number of stamens: (1) 1; (2) 2. 42. Colour of ovary: (1) white; (2) red-brown. 43. Tepal connectivity: (1) free; (2) connate. 44. Epidermis: (1) simple; (2) multiple. 45. Number of radiating epidermal cells around lithocysts: (1) 5–8; (2) 9–16. 46. Cuticular ridge abaxially: (1) present; (2) absent. 47. Occurrence of enlarged lithocysts: (1) only abaxially; (2) adaxially or abundantly adaxially and a few abaxially. 48. Crystarque cells: (1) present; (2) absent. 49. Epidermal lithocysts: (1) present; (2) absent. 50. Palisade layers: (1) single; (2) multiple (2 and more). 51. Marginal sclerenchyma: (1) present; (2) absent. 52. Vascular bundles in midrib: (1) separate bundles; (2) bundles united in two opposing arcs to a closed cylinder. 53. Pith bundles in midrib: (1) present; (2) absent. 54. Pith bundles in petiole: (1) present; (2) absent. 55. Bundle sheaths: (1) vertically transcurrent; (2) circular, not transcurrent. 56. Silicified cells in mesophyll: (1) present; (2) absent. 57. Silicified cells in epidermis: (1) present; (2) absent. 58. Glandular hairs at petiole: (1) present; (2) absent. 59. Glandular hairs at lamina: (1) present; (2) absent. 60. Stomata: (1) level to epidermis; (2) sunken. 61. Giant stomata: (1) present; (2) absent. 62. Inner stomatal ledge: (1) present; (2) absent. 63. Thickness of cuticle on adaxial lamina: (1) ≤ 1 µm; (2) > 1 µm 64. Ratio of prismatic and druse crystal in midrib: (1) prismatic > druse; (2) druse > prismatic. 65. Subepidermal sclerified layer in petiole: (1) present; (2) absent. 66. Subepidermal sclerified layer in midrib: (1) present; (2) absent.
