# Supplementary material for: A New Classification of Ficus Subsection Urostigma (Moraceae) Based on Four Nuclear DNA Markers (ITS, ETS, G3pdh, and ncpGS), Morphology and Leaf Anatomy
Source: PLoS One. 2015 Jun 24;10(6):e0128289. doi: 10.1371/journal.pone.0128289 (PMC4479584; doi:10.1371/journal.pone.0128289)
Supplement: S3 Appendix — Polymorphisms are indicated by all states presented by a comma, and inapplicable or unknown characters by “-”. etails of characters and states are also listed below. (DOCX) [file pone.0128289.s003.docx]

**S3 APPENDIX. Data matrix of morphological(1-43) and leaf anatomical(44-66) characters scored for the phylogenetic analyses and character reconstruction**. Polymorphisms are indicated by all states presented by a comma, and inapplicable or unknown characters by “-” . Details of characters and states are also listed below.

Taxon/character 1 2 3 4 5 6 7 8 9 10 11 12 13 14 15 16 17 18 19 20 21 22

*F. alongensis*1 2 2 1 1 1 1 1 1 2 2 1 1,2 1 1 1 2 1 2 1 1 1 2

*F. alongensis*2 2 2 1 1 1 1 1 1 2 2 1 1,2 1 1 1 2 1 2 1 1 1 2

*F. altissima* 2 1 2 2 1 1 1 1 2 2 1 1 1 1 1 2 1 2 1 1 2 2

*F. americana* 2 1 2 2 1 1 2 2,3 2 2 1 1 2 2 2 1 1 2 1 1 1 2

*F. arnottiana*1 2 2 1 1 1 1,2 1 1 1 1 1 2 1 2 1 2 1 1 1 1,2 1 2

*F. arnottiana*2 2 2 1 1 1 1,2 1 1 1 1 1 2 1 2 1 2 1 1 1 1,2 1 2

*F. aurea* 2 1 2 2 1 1 2 1 2 2 1 1,2 1 2 2 2 1 2 1 1 2 2

*F. benjamina* 2 1 2 2 1 2 2 1 2 2 1 1 2 2 2 1 1 2 1 1 1 2

*F. brachypoda* 2 2 2 2 1 1 2 1 2 2 1 1 2 2 2 1 1 1 1 1,2 1 2

*F. bubu* 2 1 2 2 1 2 2 1,2 2 2 1 1 1 1 1 2 1 1 1 1 1 2

*F. caulocarpa*1 2 2 1 1 1 1 1 2 2 2 1,2 1 2 2 2 2 1 1 1 1 1 2

*F. caulocarpa*2 2 2 1 1 1 1 1 2 2 2 1,2 1 2 2 2 2 1 1 1 1 1 2

*F. caulocarpa*3 2 2 1 1 1 1 1 2 2 2 1,2 1 2 2 2 2 1 1 1 1 1 2

*F. concinna*1 2 2 1 1 1 1 1 2 2 2 1 1 2 2 2 2 1 2 1 1 1 2

*F. concinna*2 2 2 1 1 1 1 1 2 2 2 1 1 2 2 2 2 1 2 1 1 1 2

*F. concinna*3 2 2 1 1 1 1 1 2 2 2 1 1 2 2 2 2 1 2 1 1 1 2

*F. concinna*4 2 2 1 1 1 1 1 2 2 2 1 1 2 2 2 2 1 2 1 1 1 2

*F. cordata*1 2 2 1 2 1 1 1 1 1 2 1 2 1 1 2 2 1 1 1 1 1 2

Taxon/character 1 2 3 4 5 6 7 8 9 10 11 12 13 14 15 16 17 18 19 20 21 22

*F. cordata*2 2 2 1 2 1 1 1 1 1 2 1 2 1 1 2 2 1 1 1 1 1 2

*F. cordata*3 2 2 1 2 1 1 1 1 1 2 1 2 1 1 2 2 1 1 1 1 1 2

*F. densifolia*1 2 2 1 2 1 1 1 1 2 2 1 1,2 1,2 1 1 2 1 1 1 1 2 2

*F. densifolia*2 2 2 1 2 1 1 1 1 2 2 1 1,2 1,2 1 1 2 1 1 1 1 2 2

*F. densifolia*3 2 2 1 2 1 1 1 1 2 2 1 1,2 1,2 1 1 2 1 1 1 1 2 2

*F. densifolia*4 2 2 1 2 1 1 1 1 2 2 1 1,2 1,2 1 1 2 1 1 1 1 2 2

*F. geniculata*1 2 2 1 1 1 1 1 1 2 2 1 1 1,2 2 2 2 1 1 1 1 1 2

*F. geniculata*2 2 2 1 1 1 1 1 1 2 2 1 1 1,2 2 2 2 1 1 1 1 1 2
*F. geniculata*3 2 2 1 1 1 1 1 1 2 2 1 1 1,2 2 2 2 1 1 1 1 1 2
*F. geniculata insignis* 2 2 1 1 1 1 1 1 2 2 1 1 1,2 2 1 2 1 1 2 1,2 1 2

*F. glaberrima siamensis*1 2 1 2 2 1 1 2 1 2 2 1 1 1 1 1 2 1 1 1 1 1 2

*F. glaberrima siamensis*2 2 1 2 2 1 1 2 1 2 2 1 1 1 1 1 2 1 1 1 1 1 2

*F. glaberrima siamensis*3 2 1 2 2 1 1 2 1 2 2 1 1 1 1 1 2 1 1 1 1 1 2

*F. henneana*1 2 2 1 1 1 1 1 2 2 2 1 1 1,2 1 2 2 1 1 1 1 1 2

*F. henneana*2 2 2 1 1 1 1 1 2 2 2 1 1 1,2 1 2 2 1 1 1 1 1 2

*F. hookeriana*1 2 2 1 2 1 1 1 2 2 2 1 1 1 1 2 2 1 1 1 1 1 1

*F. hookeriana*2 2 2 1 2 1 1 1 2 2 2 1 1 1 1 2 2 1 1 1 1 1 1

*F. ingens*1 2 2 1 2 2 1 1 1 2 2 1 1,2 1 1 1 2 1 1 1 1 1 2

*F. ingens*2 2 2 1 2 2 1 1 1 2 2 1 1,2 1 1 1 2 1 1 1 1 1 2

*F. ingens*3 2 2 1 2 2 1 1 1 2 2 1 1,2 1 1 1 2 1 1 1 1 1 2

*F. lecardii*1 2 2 1 2 1 1 1 1 2 2 1 1 1 2 2 2 1 1 1 1,2 1 2

Taxon/character 1 2 3 4 5 6 7 8 9 10 11 12 13 14 15 16 17 18 19 20 21 22

*F. lecardii*2 2 2 1 2 1 1 1 1 2 2 1 1 1 2 2 2 1 1 1 1,2 1 2

*F. madagascariensis* 1,2 2 1 2 1 1 1 1 2 2 1 1 1,2 2 2 2 1 1 1 1 1 2

*F. maxima* 2 2 2 2 1 2 2 1 2 2 1 1 1 1 2 2 2 2 2 1 2 2

*F. menabeensis* 2 1 2 2 1 2 2 1 2 2 1 1 1 2 2 2 1 2 1 2 1 2

*F. middletonii* 2 2 1 1 1 1 1 1 2 2 1 1 1 1 1 2 1 1 1 1,2 1 2

*F. orthoneura*1 1 2 1 2 1 1 1 2 2 2 1 1 2 1 2 2 1 1 1 1 1 1

*F. orthoneura*2 1 2 1 2 1 1 1 2 2 2 1 1 2 1 2 2 1 1 1 1 1 1

*F. orthoneura*3 1 2 1 2 1 1 1 2 2 2 1 1 2 1 2 2 1 1 1 1 1 1

*F. pleurocarpa* 2 2 2 2 2 1 2 1 2 2 1 1 2 2 2 1 1 2 1 2 2 2

*F. prasinicarpa*1 2 2 1 1 1 2 1 1 2 2 1 1,2 1 2 1 2 1 1 1 1 1 2

*F. prasinicarpa*2 2 2 1 1 1 2 1 1 2 2 1 1,2 1 2 1 2 1 1 1 1 1 2

*F. prolixa*1 2 2 1 1 1 1 1 1 2 2 1 1,2 1 2 1 2 1 1 1,2 1,2 1 2

*F. prolixa*2 2 2 1 1 1 1 1 1 2 2 1 1,2 1 2 1 2 1 1 1,2 1,2 1 2

*F. pseudoconcinna* 2 2 1 1 1 1 1 2 2 2 1 1 1 2 2 2 1 1 1 1 1 2

*F. religiosa*1 2 2 1 1 1 1 1,2 1 1 1 1 1,2 1 1 1 2 1 1 1 1 1 2

*F. religiosa*2 2 2 1 1 1 1 1,2 1 1 1 1 1,2 1 1 1 2 1 1 1 1 1 2

*F. religiosa*3 2 2 1 1 1 1 1,2 1 1 1 1 1,2 1 1 1 2 1 1 1 1 1 2

*F. rumphii*1 2 2 1 2 1 2 1 1 2 2 1 2 1 1 1 2 1 1 1 1 2 2

*F. rumphii*2 2 2 1 2 1 2 1 1 2 2 1 2 1 1 1 2 1 1 1 1 2 2

*F. rumphii cf.*  2 2 2 2 1 2 1 1 2 2 1 2 1 1 1 2 1 2 1 2 1 2

*F. salicifolia*1 2 2 1 2 1 1 1 1 2 2 2 1 2 2 2 2 1 1 1 1,2 1 2

Taxon/character 1 2 3 4 5 6 7 8 9 10 11 12 13 14 15 16 17 18 19 20 21 22

*F. salicifolia*2 2 2 1 2 1 1 1 1 2 2 2 1 2 2 2 2 1 1 1 1,2 1 2

*F. subpisocarpa pubipoda*1 2 2 1 1 1 2 1 2 2 2 1 1 1 2 2 2 1 1 2 2 1 1

*F. subpisocarpa pubipoda*2 2 2 1 1 1 2 1 2 2 2 1 1 1 2 2 2 1 1 2 2 1 1

*F. superba*1 2 2 1 1 1 1 1 2 2 2 1 1 1 1 2 2 1 1 1 1 2 1

*F. superba*2 2 2 1 1 1 1 1 2 2 2 1 1 1 1 2 2 1 1 1 1 2 1

*F. tonduzii* 2 2 2 2 1 1 2 1 2 2 1 1 1 1 2 2 2 1 2 1 2 2

*F. tsjakela* 2 2 1 1 1 1 1 1 2 2 1 1 2 2 2 2 1 1 1 1 1 2

*F. verruculosa*1 1 2 1 2 1,2 1 2 1,2 2 2 1 1,2 2 2 2 2 1 2 1 1,2 1 2

*F. verruculosa*2 1 2 1 2 1,2 1 2 1,2 2 2 1 1,2 2 2 2 2 1 2 1 1,2 1 2

*F. virens*1 2 2 1 1 1 1 1 1 1 2 1 1 1 1 1 2 1 1 1 1 1 1

*F. virens*2 2 2 1 1 1 1 1 1 2 2 1 1 1,2 1 1 2 1 1 1 1 1 1

*F. virens*3 2 2 1 1 1 1 1 1 2 2 1 1 1,2 1 1 2 1 1 1 1 1 1

*F. virens*4 2 2 1 1 1 2 1 1 2 2 1 1 1,2 2 2 1 1 1 1 1 2 2

*F. virens*5 2 2 1 1 1 2 1 1 2 2 1 1 1,2 2 2 1 1 1 1 1 2 2

*F. virens glabella*1 2 2 1 1 1 1 1 3 2 2 1 1 1,2 2 2 2 1 1 1 1 1 1

*F. virens glabella*2 2 2 1 1 1 1 1 3 2 2 1 1 1,2 2 2 2 1 1 1 1 1 1

Taxon/character 23 24 25 26 27 28 29 30 31 32 33 34 35 36 37 38 39 40 41 42 43 44

*F. alongensis*1 1 1 2 2 1 1 1 2 1 1 1 1 - 1 1 1 2 1 1 2 1,2 1

*F. alongensis*2 1 1 2 2 1 1 1 2 1 1 1 1 - 1 1 1 2 1 1 2 1,2 1

*F. altissima* 1 1 2 2 1 1 2 1,2 1 1 2 1 1 1 1 1 2 2 1 2 1 -

Taxon/character 23 24 25 26 27 28 29 30 31 32 33 34 35 36 37 38 39 40 41 42 43 44

*F. americana* 1 1 1 1 2 1 1 1 1 1 1 1 1 1 1 1 2 2 1 - 1 -

*F. arnottiana*1 1 1 2 1 2 1 1 2 1 1 1 1 - 1 1 1 2 2 1 2 2 2

*F. arnottiana*2 1 1 2 1 2 1 1 2 1 1 1 1 - 1 1 1 2 2 1 2 2 2

*F. aurea* 1 1 1 2 1 1 1,2 1 1 2 1 1 1 1 1 1 - 2 1 2 1 -

*F. benjamina* 1 1 2 2 1 1 2 2 1 1 1 1 1 1 1 1 1 2 1 2 1 -

*F. brachypoda* 1 1 1,2 2 1 1 1 2 2 1 2 3 1 1 1 1 2 2 1 2 1 -

*F. bubu* 1 1 2 1 2 1 1 2 1 1 2 1 1 3 1 1 2 2 1 2 1 -

*F. caulocarpa*1 1 1 1 1 2 1 1 2 1 1 1 1 2 1 1 1 1 1 1 2 1,2 1

*F. caulocarpa*2 1 1 1 1 2 1 1 2 1 1 1 1 2 1 1 1 1 1 1 2 1,2 1

*F. caulocarpa*3 1 1 1 1 2 1 1 2 1 1 1 1 2 1 1 1 1 1 1 2 1,2 1

*F. concinna*1 1 1 2 1 2 1 1 2 2 1 1 1 2 1 1 1 2 1 1 2 2 1

*F. concinna*2 1 1 2 1 2 1 1 2 2 1 1 1 2 1 1 1 2 1 1 2 2 1

*F. concinna*3 1 1 2 1 2 1 1 2 2 1 1 1 2 1 1 1 2 1 1 2 2 1

*F. concinna*4 1 1 2 1 2 1 1 2 2 1 1 1 2 1 1 1 2 1 1 2 2 1

*F. cordata*1 1 1 2 1 1 1 2 2 1 1 1 1 1 1 1 1,2 2 1 1 2 1 1

*F. cordata*2 1 1 2 1 1 1 2 2 1 1 1 1 1 1 1 1,2 2 1 1 2 1 1

*F. cordata*3 1 1 2 1 1 1 2 2 1 1 1 1 1 1 1 1,2 2 1 1 2 1 1

*F. densifolia*1 1 1 1 2 1 1 2 2 1 2 1 1 - 1 1 1 2 2 1 2 1 1

*F. densifolia*2 1 1 1 2 1 1 2 2 1 2 1 1 - 1 1 1 2 2 1 2 1 1

*F. densifolia*3 1 1 1 2 1 1 2 2 1 2 1 1 - 1 1 1 2 2 1 2 1 1

*F. densifolia*4 1 1 1 2 1 1 2 2 1 2 1 1 - 1 1 1 2 2 1 2 1 1

Taxon/character 23 24 25 26 27 28 29 30 31 32 33 34 35 36 37 38 39 40 41 42 43 44

*F. geniculata*1 1 1 1 1 2 1 1,2 2 1 1 1 1 2 1 1 1 1 1 1 2 2 1

*F. geniculata*2 2 2 1 1 1 1 1 2 1 1 1 2 2 2 2 1 2 2 2 1 1 1

*F. geniculata*3 2 2 1 1 1 1 1 2 1 1 1 2 2 2 2 1 2 2 2 1 1 1

*F. geniculata insignis* 1 2 1 1 2 2 1,2 2 1 1 1 1 2 1 1 1 1 1 1 2 2 1

*F. glaberrima siamensis*1 1 1 2 2 1 1 1 2 2 1 1 1 1 1 1 1 2 2 1 2 1,2 2

*F. glaberrima siamensis*2 1 1 2 2 1 1 1 2 2 1 1 1 1 1 1 1 2 2 1 2 1,2 2

*F. glaberrima siamensis*3 1 1 2 2 1 1 1 2 2 1 1 1 1 1 1 1 2 2 1 2 1,2 2

*F. henneana*1 1 1 2 2 1 1 1 2 2 1 2 1 2 1 2 1 2 1 1 2 1 1

*F. henneana*2 1 1 2 2 1 1 1 2 2 1 2 1 2 1 2 1 2 1 1 2 1 1

*F. hookeriana*1 1 1 1 2 1 1 2 - 1 2 2 1 - 2 2 1 2 2 1 2 1 2

*F. hookeriana*2 1 1 1 2 1 1 2 - 1 2 2 1 - 2 2 1 2 2 1 2 1 2

*F. ingens*1 1 1 2 2 1 1 1 2 1 1 1,2 1 1 1 1 1,2 1 1 1 2 1 1

*F. ingens*2 1 1 2 2 1 1 1 2 1 1 1,2 1 1 1 1 1,2 1 1 1 2 1 1

*F. ingens*3 1 1 2 2 1 1 1 2 1 1 1,2 1 1 1 1 1,2 1 1 1 2 1 1

*F. lecardii*1 1 1 2 2 1 1 1 2 1 1 1 1 1 1 1 1,2 1 1 1 2 1 1

*F. lecardii*2 1 1 2 2 1 1 1 2 1 1 1 1 1 1 1 1,2 1 1 1 2 1 1

*F. madagascariensis* 1 1 1 2 1 1 2 2 1 1 1 1 - 1 1 1 2 1 1 2 1 1

*F. maxima* 1 1 1 2 1 1 1 2 1 1 2 1 - 1 1 2 2 2 2 2 1 -

*F. menabeensis* 1 1 2 2 1 1 2 2 1 1 1 1 1 1 1 1 - 2 1 1 1 -

*F. middletonii* 1 1 1 1 1 1 1 2 1 1 1 1 1 1 1 1 1 1 1 2 1 1

*F. orthoneura*1 1 1 1 1 1 1 1 2 1 1 1,2 1 - 1 1,2 1 2 2 1 2 1 2

Taxon/character 23 24 25 26 27 28 29 30 31 32 33 34 35 36 37 38 39 40 41 42 43 44

*F. orthoneura*2 1 1 1 1 1 1 1 2 1 1 1,2 1 - 1 1,2 1 2 2 1 2 1 2

*F. orthoneura*3 1 1 1 1 1 1 1 2 1 1 1,2 1 - 1 1,2 1 2 2 1 2 1 2

*F. pleurocarpa* 1 2 1 2 1 1 1 2 1 1 2 1 1 1 1 1 2 2 1 2 1 -

*F. prasinicarpa*1 1 1 2 1 1 1 1 2 1 1 1 1,2 2 1 1 1 2 1 1 2 1 1

*F. prasinicarpa*2 1 1 2 1 1 1 1 2 1 1 1 1,2 2 1 1 1 2 1 1 2 1 1

*F. prolixa*1 1 1 1 1 1 1 1 2 1 1 1 1,2 2 1 1 1 1 2 1 2 1,2 1

*F. prolixa*2 1 1 1 1 1 1 1 2 1 1 1 1,2 2 1 1 1 1 2 1 2 1,2 1

*F. pseudoconcinna* 1 1 1 2 1 1 1 2 1 1 1 1 2 1 1 1 2 1 1 2 1 1

*F. religiosa*1 1 1 1,2 2 1 1 2 2 1 2 1 1 2 1 1 1 2 1 1 2 1 1

*F. religiosa*2 1 1 1,2 2 1 1 2 2 1 2 1 1 2 1 1 1 2 1 1 2 1 1

*F. religiosa*3 1 1 1,2 2 1 1 2 2 1 2 1 1 2 1 1 1 2 1 1 2 1 1

*F. rumphii*1 1 1 1 2 1 1 2 1,2 1 1 1 2 2 1 1 1 2 2 1 1 1 2

*F. rumphii*2 1 1 1 2 1 1 2 1,2 1 1 1 2 2 1 1 1 2 2 1 1 1 2

*F. rumphii cf.*  1 1 1 2 1 1 2 2 1 1 1 2 2 1 1 1 2 2 1 1 1 2

*F. salicifolia*1 1 1 1 1 1 1 1 2 1 1 1,2 1 2 1 1 1,2 1 1 1 2 1 1

*F. salicifolia*2 1 1 1 1 1 1 1 2 1 1 1,2 1 2 1 1 1,2 1 1 1 2 1 1

*F. subpisocarpa pubipoda*1 1 1 1 1 2 1 1 2 2 1 1,2 1,2 2 1 1 1 1 1 1 2 2 1

*F. subpisocarpa pubipoda*2 1 1 1 1 2 1 1 2 2 1 1,2 1,2 2 1 1 1 1 1 1 2 2 1

*F. superba*1 1 2 1 1 2 1 1 2 2 1 2 2 2 1 1 1 2 1 1 2 1,2 1

*F. superba*2 1 2 1 1 2 1 1 2 2 1 2 2 2 1 1 1 2 1 1 2 1,2 1

Taxon/character 23 24 25 26 27 28 29 30 31 32 33 34 35 36 37 38 39 40 41 42 43 44

*F. tonduzii* 1 1 1 2 1 1 1 2 1 1 2 1 - 1 1 1 2 2 2 2 1 -

*F. tsjakela* 1 1 1 1 2 1 2 2 1 1 1 1 - 1 1 1 2 1 1 2 1 1

*F. verruculosa*1 1 1 1 1 2 1 1 2 1 1 1 1 1 1 1 1 2 1 1 2 1 1

*F. verruculosa*2 1 1 1 1 2 1 1 2 1 1 1 1 1 1 1 1 2 1 1 2 1 1

*F. virens*1 1 1 1 1 2 1 1 2 1 1 1 1 2 1 1 1 1 1 1 2 2 1

*F. virens*2 1 1 2 1 1 1 1,2 2 1 1 1,2 1 2 1 1 2 1 1 1 2 1 1

*F. virens*3 1 1 2 1 1 1 1,2 2 1 1 1,2 1 2 1 1 2 1 1 1 2 1 1

*F. virens*4 1 1 2 2 1 1 2 2 1 1 1 2 1 2 1 1 2 2 1 1 1 2

*F. virens*5 1 1 2 2 1 1 2 2 1 1 1 2 1 2 1 1 2 2 1 1 1 2

*F. virens glabella*1 2 1 1 1 2 1 1,2 2 1 1 1 1 2 1 1 1 1 1 1 2 2 1

*F. virens glabella*2 2 1 1 1 2 1 1,2 2 1 1 1 1 2 1 1 1 1 1 1 2 2 1

Taxon/character 45 46 47 48 49 50 51 52 53 54 55 56 57 58 59 60 61 62 63 64 65 66

*F. alongensis*1 2 2 1 1 2 1 1 1 2 2 1 2 2 1 2 1 2 2 2 1 1 1
*F. alongensis*2 2 2 1 1 2 1 1 1 2 2 1 2 2 1 2 1 2 2 2 1 1 1

*F. altissima* - - - - - - - - - - - - - - - - - - - - - -
*F. americana* - - - - - - - - - - - - - - - - - - - - - -
*F. arnottiana*1 1 2 2 2 2 2 1,2 2 1 1 1 2 2 2 2 1 2 1 1 2 2 2
*F. arnottiana*2 1 2 2 2 2 2 1,2 2 1 1 1 2 2 2 2 1 2 1 1 2 2 2
*F. aurea* - - - - - - - - - - - - - - - - - - - - - -

Taxon/character 45 46 47 48 49 50 51 52 53 54 55 56 57 58 59 60 61 62 63 64 65 66

*F. benjamina* - - - - - - - - - - - - - - - - - - - - - -
*F. brachypoda* - - - - - - - - - - - - - - - - - - - - - -
*F. bubu* - - - - - - - - - - - - - - - - - - - - - -
*F. caulocarpa*1 2 2 1 1,2 2 2 1 2 1 1 1 1 2 1 2 1 2 2 2 1 1 1
*F. caulocarpa*2 2 2 1 1,2 2 2 1 2 1 1 1 1 2 1 2 1 2 2 2 1 1 1
*F. caulocarpa*3 2 2 1 1,2 2 2 1 2 1 1 1 1 2 1 2 1 2 2 2 1 1 1
*F. concinna*1 2 2 1 1 2 2 1 2 1 1 1 1 2 1 2 1 2 1 2 1 1 1
*F. concinna*2 2 2 1 1 2 2 1 2 1 1 1 1 2 1 2 1 2 1 2 1 1 1
*F. concinna*3 2 2 1 1 2 2 1 2 1 1 1 1 2 1 2 1 2 1 2 1 1 1
*F. concinna*4 2 2 1 1 2 2 1 2 1 1 1 1 2 1 2 1 2 1 2 1 1 1
*F. cordata*1 2 2 1 1 1,2 2 2 2 1 1 2 1,2 2 2 2 2 2 2 1 1 1 1
*F. cordata*2 2 2 1 1 1,2 2 2 2 1 1 2 1,2 2 2 2 2 2 2 1 1 1 1
*F. cordata*3 2 2 1 1 1,2 2 2 2 1 1 2 1,2 2 2 2 2 2 2 1 1 1 1
*F. densifolia*1 1 2 1 2 2 2 1 2 2 1 1 2 2 2 2 1 1 2 2 1 2 1
*F. densifolia*2 1 2 1 2 2 2 1 2 2 1 1 2 2 2 2 1 1 2 2 1 2 1
*F. densifolia*3 1 2 1 2 2 2 1 2 2 1 1 2 2 2 2 1 1 2 2 1 2 1
*F. densifolia*4 1 2 1 2 2 2 1 2 2 1 1 2 2 2 2 1 1 2 2 1 2 1
*F. geniculata*1 2 2 1 1 1 2 1 2 1 1 1 2 2 2 2 1 2 2 2 1 1 1
*F. geniculata*2 2 2 1 1 1 2 1 2 1 1 1 2 2 2 2 1 2 2 2 1 1 1
*F. geniculata*3 2 2 1 1 1 2 1 2 1 1 1 2 2 2 2 1 2 2 2 1 1 1

Taxon/character 45 46 47 48 49 50 51 52 53 54 55 56 57 58 59 60 61 62 63 64 65 66

*F. geniculata insignis* 2 2 1 1 2 2 1 2 1 1 1 2 2 1 2 1 1 2 1 1 1 1
*F. glaberrima siamensis*1 1 2 2 2 2 1 1 2 1 1 1 2 2 2 2 1 1 2 1,2 2 2 2
*F. glaberrima siamensis*2 1 2 2 2 2 1 1 2 1 1 1 2 2 2 2 1 1 2 1,2 2 2 2
*F. glaberrima siamensis*3 1 2 2 2 2 1 1 2 1 1 1 2 2 2 2 1 1 2 1,2 2 2 2
*F. henneana*1 2 2 1 2 1 2 1 2 1 1 1 2 2 2 2 1 2 2 1 1 2 1
*F. henneana*2 2 2 1 2 1 2 1 2 1 1 1 2 2 2 2 1 2 2 1 1 2 1
*F. hookeriana*1 1 1 1 1,2 1 1,2 2 2 1 1 1 2 2 2 2 1 2 2 2 1 2 1
*F. hookeriana*2 1 1 1 1,2 1 1,2 2 2 1 1 1 2 2 2 2 1 2 2 2 1 2 1
*F. ingens*1 2 2 1 1 1 2 1 2 1 1 1 1,2 2 1,2 1,2 1 2 2 1 1 1 1
*F. ingens*2 2 2 1 1 1 2 1 2 1 1 1 1,2 2 1,2 1,2 1 2 2 1 1 1 1
*F. ingens*3 2 2 1 1 1 2 1 2 1 1 1 1,2 2 1,2 1,2 1 2 2 1 1 1 1
*F. lecardii*1 1 2 1 2 1 1 2 2 1 1 1 2 2 2 2 2 1 2 1 1 2 1
*F. lecardii*2 1 2 1 2 1 1 2 2 1 1 1 2 2 2 2 2 1 2 1 1 2 1
*F. madagascariensis* - - 1 - - - - - - - - - - - - - - - - - - -
*F. maxima* - - - - - - - - - - - - - - - - - - - - - -

*F. menabeensis* - - - - - - - - - - - - - - - - - - - - - -
*F. middletonii* 2 2 1 2 2 2 1 2 1 1 1 2 2 1 1 1 2 1,2 1 1 1 1
*F. orthoneura*1 1 1 1 2 2 1,2 1 1 1 1 1 2 2 2 2 1 2 2 2 1 1 1
*F. orthoneura*2 1 1 1 2 2 1,2 1 1 1 1 1 2 2 2 2 1 2 2 2 1 1 1
*F. orthoneura*3 1 1 1 2 2 1,2 1 1 1 1 1 2 2 2 2 1 2 2 2 1 1 1

Taxon/character 45 46 47 48 49 50 51 52 53 54 55 56 57 58 59 60 61 62 63 64 65 66

*F. pleurocarpa* - - - - - - - - - - - - - - - - - - - - - -
*F. prasinicarpa*1 1 2 1 1,2 1,2 1 1 2 1 1 1 1 1 2 2 1 2 2 1 1 1 1
*F. prasinicarpa*2 1 2 1 1,2 1,2 1 1 2 1 1 1 1 1 2 2 1 2 2 1 1 1 1
*F. prolixa*1 2 2 1 1,2 1,2 1 1 2 1 1 1 1 1,2 1 2 1 2 1,2 1 1 1 1
*F. prolixa*2 2 2 1 1,2 1,2 1 1 2 1 1 1 1 1,2 1 2 1 2 1,2 1 1 1 1
*F. pseudoconcinna* 1 2 1 1 1,2 1 1 2 1 1 1 1 1 2 2 1 2 2 2 1 1 1
*F. religiosa*1 2 2 1 1 1,2 2 1 2 1 1 2 1 1 2 2 1 1 2 1 1 1 1
*F. religiosa*2 2 2 1 1 1,2 2 1 2 1 1 2 1 1 2 2 1 1 2 1 1 1 1
*F. religiosa*3 2 2 1 1 1,2 2 1 2 1 1 2 1 1 2 2 1 1 2 1 1 1 1
*F. rumphii*1 1 2 2 2 2 2 1 2 1 1 1 2 2 1 2 1 1 2 2 2 2 2
*F. rumphii*2 1 2 2 2 2 2 1 2 1 1 1 2 2 1 2 1 1 2 2 2 2 2
*F. rumphii cf*. 1 2 2 2 2 2 1 2 1 1 1 1 2 1 1 1 2 2 2 2 2 2

*F. salicifolia*1 1 2 1 2 1 2 1 2 1,2 1 1 2 2 1 2 2 1 1 1 1 2 2
*F. salicifolia*2 1 2 1 2 1 2 1 2 1,2 1 1 2 2 1 2 2 1 1 1 1 2 2
*F. subpisocarpa pubipoda*1 1 2 1 1 1 1 1 2 1 1 1 2 2 1 2 1 2 1 1 1 1 1
*F. subpisocarpa pubipoda*2 1 2 1 1 1 1 1 2 1 1 1 2 2 1 2 1 2 1 1 1 1 1
*F. superba*1 1 2 1 1 2 1 1 2 1 1 1 2 2 2 2 1 2 2 1 1 1 1
*F. superba*2 1 2 1 1 2 1 1 2 1 1 1 2 2 2 2 1 2 2 1 1 1 1

*F. tonduzii* - - - - - - - - - - - - - - - - - - - - - -
*F. tsjakela* 1 1 1 1,2 1,2 2 2 2 1 1 2 1,2 2 2 2 2 1 2 2 1 1 1

Taxon/character 45 46 47 48 49 50 51 52 53 54 55 56 57 58 59 60 61 62 63 64 65 66

*F. verruculosa*1 1 2 1 1,2 1,2 1 2 2 1,2 1 1 1 1 2 2 1,2 2 2 1 1 2 2
*F. verruculosa*2 1 2 1 1,2 1,2 1 2 2 1,2 1 1 1 1 2 2 1,2 2 2 1 1 2 2
*F. virens*1 2 2 1 1 1 2 1 2 1 1 1 1 1 2 2 1 2 2 1 1 1 1
*F. virens*2 2 2 1 1 1 2 1 2 1 1 1 1 1 2 2 1 2 2 1 1 1 1
*F. virens*3 2 2 1 1 1 2 1 2 1 1 1 1 1 2 2 1 2 2 1 1 1 1
*F. virens*4 1 2 2 2 2 2 1 2 1 1 1 1,2 1,2 2 2 2 1 2 2 2 2 1
*F. virens*5 1 2 2 2 2 2 1 2 1 1 1 1,2 1,2 2 2 2 1 2 2 2 2 1
*F. virens glabella*1 2 2 1 1 1 2 1 2 1 1 1 2 1 2 2 1 2 2 2 1 1 1

*F. virens glabella*2 2 2 1 1 1 2 1 2 1 1 1 2 1 2 2 1 2 2 2 1 1 1
